# Nucleophilic Fluorination of a Secondary Alkyl Bromide with KF(18‐Crown‐6) and Bulky Diols: Microsolvation Causes Chemoselectivity Inversion in the Free Energy Profile

**DOI:** 10.1002/cplu.202500257

**Published:** 2025-07-01

**Authors:** Luís F. Resende, Eloah P. Ávila, Josefredo R. Pliego

**Affiliations:** ^1^ Departamento de Ciências Naturais Universidade Federal de São João del‐Rei 36301‐160 São João del‐Rei Minas Gerais Brazil

**Keywords:** crown compounds, density functional calculations, fluorine, hydrogen bonds, solvent effects

## Abstract

Fluoride ion solvated in polar aprotic solvents works like bases and reacts with secondary alkyl bromide substrates mainly via E2 reactions, with minor formation of S_N_2 fluorination products. The use of KF combined with crown ether increases the S_N_2 yield. However, secondary substrates remain a challenge for nucleophilic fluorination. It has recently been demonstrated that crown ethers combined with the fluorinated *tert*‐butyl alcohol (TBOH) TBOH‐F3 can increase the KF salt reactivity and selectivity toward S_N_2 reactions. These observations can be explained by the microsolvation of the fluoride ion with hydrogen bond donor species. This study explores computationally the effect of bulky diols in the reaction of KF(18‐crown‐6) with 2‐bromopropane as a model substrate. This study investigates the microsolvation by TBOH, TBOH‐F3, and the bulky diols pinacol and BDMb‐F6, aimed at evaluating their effect on the S_N_2:E2 product ratio. Considering the competitive pathways, results show that TBOH is the least effective, while bulky alcohol TBOH‐F3, pinacol, and BDMb‐F6 favor the S_N_2 pathway by 0.5, 1.6, and 5.6 kcal mol^−1^. Thus, the study's findings indicate that pinacol and especially the BDMb‐F6 diol are highly promising alcohols for achieving greater S_N_2:E2 selectivity in nucleophilic substitution reactions utilizing KF(18‐crown‐6).

## Graphical abstract

The combination of crown ether with strong hydrogen bond donor bulky diols enhances reactivity and S_N_2 selectivity in challenging fluorination reactions of secondary alkyl bromide substrates.

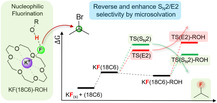

## 1 Introduction

While naturally occurring organofluorine compounds are rare, with only ≈30 identified in plants and microorganisms,^[^ ^1^ ^]^ the synthetic organofluorine market size has experienced significant growth. According to Verified Market Reports, it reached USD 6.5 billion in 2024^[^ ^2^ ^]^ and the global demand for fluorochemicals was projected to exceed 5300 kilotons in this same year.^[^ ^3^ ^]^ The growing demand is driven by the widespread application of organofluorines in diverse fields. Notably, an estimated 20% of pharmaceuticals and 30%–40% of agrochemicals contain fluorine.^[^ ^4^ ^]^ Furthermore, they are applied for components in electronic/optoelectronic devices,^[^ ^5^ ^]^ lithium‐ion batteries,^[^ ^6^ ^]^ fragrances,^[^ ^7^ ^]^ polymers,^[^ ^3^ ^]^ positron emission tomography,^[^ ^8^ ^]^ and various other materials.^[^ ^9^ ^]^ The unique ability of C—F bonds to modify physicochemical properties and biological activity responses of bioactive molecules has driven the development of advanced monofluorination methodologies over the past two decades,^[^ ^10^, ^11^, ^12^, ^13^, ^14^, ^15^, ^16^, ^17^, ^18^, ^19^, ^20^, ^21^, ^22^, ^23^, ^24^, ^25^ ^]^ including asymmetric catalysis.^[^ ^26^, ^27^ ^]^

**Figure 1:**
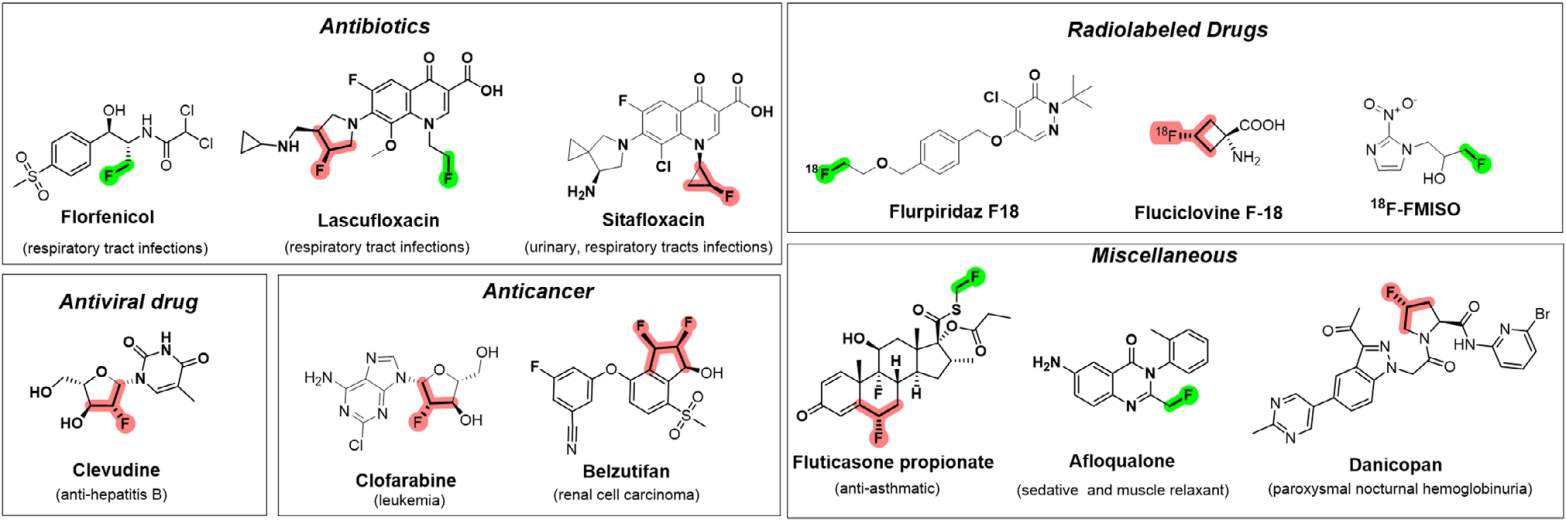
FDA‐approved drugs containing primary and secondary alkyl fluorides.

Late‐stage fluorination is becoming an attractive approach for the synthesis of novel pharmaceuticals.^[^ ^28^, ^29^, ^30^ ^]^ In the case of nucleophilic fluorination of aliphatic compounds, the reaction leads to the building of compounds containing —CHF and —CH_2_F groups. Some pharmaceuticals with these functional groups are presented in **Figure** **1**. For example, these skeletons are present in broad‐spectrum antibiotics such as florfenicol, lascufloxacin, and sitafloxacin,^[^ ^31^, ^32^, ^33^ ^]^ and we can observe both primary and secondary fluoride as β‐fluoroamine moieties. The primary alkyl fluoride group can also be found as a fluorohydrin, another important motif, in the radiolabeled drug ^18^ F‐FMISO.^[^ ^34^ ^]^ Secondary alkyl moieties can be found as enantiopure *trans*‐β‐fluorohydrins‐containing drugs Clevudine^[^ ^35^ ^]^ and Clofarabine,^[^ ^36^ ^]^ with antiviral and anticancer uses, respectively. Another interesting example is Belzutifan,^[^ ^37^ ^]^ a chemotherapy drug presenting *cis*‐β‐fluorohydrin group. More diverse structures, such as fluorinated amino acid skeletons, are present in the radiopharmaceutical fluciclovine F18^[^ ^38^ ^]^ and Danicopan, a recently food and drug administration (FDA)‐approved first‐in‐class drug for paroxysmal nocturnal hemoglobinuria. Finally, these alkyl fluoride units can be found in afloqualone,^[^ ^39^ ^]^ a nonbenzodiazepine skeletal muscle relaxant; flurpiridaz F18^[^ ^40^ ^]^ used in diagnostic imaging for myocardial ischemia; and fluticasone propionate,^[^ ^41^ ^]^ widely used as an antiasthmatic agent and chronic rhinosinusitis treatment in adults. These examples underscore the significance of aliphatic fluorides in the pharmaceutical sector.

Condensed‐phase reactions involving the fluoride ion, such as aliphatic nucleophilic substitutions, are extremely challenging due to the high basicity of the F^−^ anion.^[^ ^42^ ^]^ For example, the use of tetrabutyl ammonium fluoride (TBAF) in polar aprotic solvents leads to considerable competition with E2 reactions, with the formation of elimination products (50% E2 yield for 1‐bromooctane).^[^ ^43^, ^44^ ^]^ Aimed at overcoming this limitation, hydrogen bond donor species such as *tert*‐butyl alcohol (TBOH) or diarylureas can be added to the TBAF to reduce the basicity of the fluoride ion and to improve the S_N_2 yield.^[^ ^45^, ^46^, ^47^, ^48^, ^49^ ^]^ The drawback is that this microsolvation^[^ ^45^ ^]^ also results in a decrease in the reaction rate. Thus, controlling the solvent effect is critical for modulating reactivity and selectivity.

**Figure 2:**
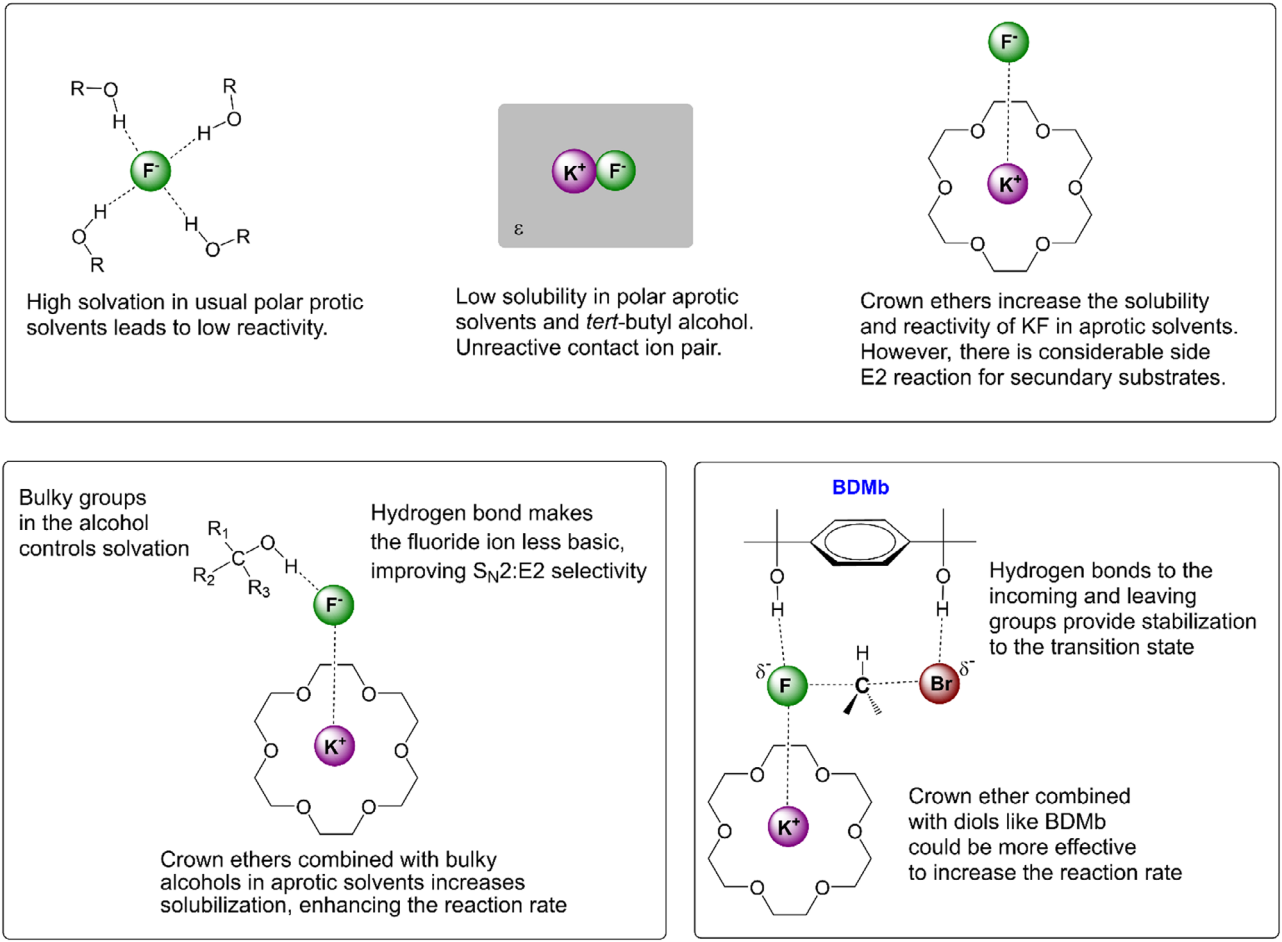
Reactivity of the fluoride ion under different conditions.

In 2006, Kim and coworkers reported that some protic solvents, such as TBOH and *tert*‐amyl alcohol, exhibit superior efficacy in the nucleophilic fluorination of alkyl mesylates with CsF compared to polar aprotic solvents.^[^ ^50^ ^]^ Posterior theoretical calculations, including molecular dynamics simulations, have supported the view that these reactions take place via a CsF contact ion pair,^[^ ^51^ ^]^ an important concept for describing this kind of reaction. When the less soluble KF salt was used, only trace amounts of substitution products were observed.^[^ ^50^ ^]^ Owing to the high lattice enthalpy of KF (194.1 kcal mol^−1^),^[^ ^52^ ^]^ controlling the solubility and activation of the fluoride ion in nucleophilic fluorination becomes a solvation engineering problem (**Figure** **2**). While usual polar protic solvents contribute to solubilization, they retard the S_N_2 reaction kinetics due to the degree of solvation, particularly for small anions (Δ*G* ^*^_solv_(F^−^) = −117.0 kcal mol^−1^ in H_2_O).^[^ ^53^ ^]^ Although polar aprotic solvents are desirable for usual nucleophilic substitutions, these solvents do not provide sufficient electrostatic interactions to overcome the crystal lattice potential energy for complete solubilization ((Δ*G* ^*^_solv_(F^−^) = −88.0 kcal mol^−1^ in acetonitrile).^[^ ^53^ ^]^ A solution to this problem is the use of crown ethers and cryptands.^[^ ^42^ ^]^ Thus, in 1974, Liotta and Harris introduced the concept of “naked anions” by utilizing KF with 18‐crown‐6 ether (18C6) as a coordination species in aprotic organic solvents such as benzene and acetonitrile.^[^ ^54^ ^]^ The idea was to make the fluoride ion soluble and “free” in solution, able to promote fluorination. However, apolar solvents like benzene are not able to separate ion pairs.^[^ ^42^ ^]^ Based on this view, Pliego and Riveros first modeled the reaction of KF(18C6) with ethyl bromide in a toluene solution using the ion pair concept, explaining the experimental findings.^[^ ^55^ ^]^ Posterior molecular dynamics calculations in acetonitrile solution by Pliego have pointed out that even in this solvent, the fluoride ion is not “free” or “naked.” Rather, it forms a contact ion pair with the potassium ion coordinated by 18‐crown‐6.^[^ ^56^ ^]^ Very recently, experimental studies by Kim and coworkers have provided further support for the ion pair concept.^[^ ^57^ ^]^ In the past years, various theoretical and experimental studies on structured microenvironments have been reported, including ionic liquids, cryptands, and functionalized crown ethers.^[^ ^58^, ^59^, ^60^, ^61^, ^62^, ^63^, ^64^, ^65^, ^66^, ^67^, ^68^ ^]^

The idea of controlling microsolvation in crown ether‐catalyzed reactions was more explored in 2020, with the introduction of the bulky diol 1,4‐bis(2‐hydroxy‐2‐propyl)benzene (BDMb, Figure 2). The effect of this diol was theoretically and experimentally studied in the reaction between KF(18C6) and a primary alkyl bromide in acetonitrile solvent.^[^ ^11^ ^]^ Theoretical studies have pointed out that BDMb can work via double hydrogen bonding to the incoming fluoride ion and the leaving bromide ion (Figure 2). In addition, some experiments have supported that BDMb combined with 18C6 is able to promote effective fluorination (46% yield) and high S_N_2:E2 selectivity (94:6) in mild reaction conditions (82 °C, 24 h).^[^ ^11^ ^]^ More recently, the combination of 18C6 with fluorinated bulky alcohols was introduced by Pliego´s group.^[^ ^10^, ^69^ ^]^ Using computational modeling to shed light on the process and extensive experimental studies, they reported the fluorination of (3‐bromopropoxy)benzene using KF as a reactant and 18C6 combined with TBOH‐F3 alcohol, achieving 89% conversion in a short reaction time of 6 h at a mild temperature of 82 °C. It was reported that there was a 70% yield for nucleophilic fluorination and minimal formation (5%) of E2 product, with a 14% yield of hydrolysis (S_N_2:E2 ratio of 93:7).^[^ ^10^ ^]^ In the case of the more challenging secondary substrates 1‐(3‐bromobutyl)‐4‐methoxybenzene, it was reported total conversion of the substrate, with 44% yield via S_N_2% and 56% via E2 under 82 °C and 18 h of reaction time.^[^ ^10^ ^]^ Interestingly, no hydrolysis product from the water present in the medium was observed.

**Figure 3:**
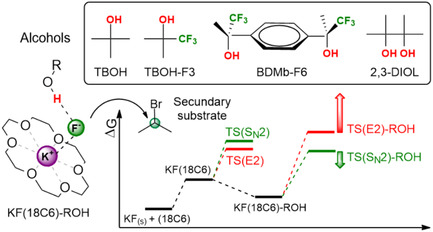
Schematic representation of the KF(18C6) complex microsolvated by different alcohols, ROH, for the S_N_2 nucleophilic fluorination reaction with 2‐bromopropane, and how the microsolvated environment can increase S_N_2:E2 selectivity, according to the Gibbs free energy profile, Δ*G*.

Based on the reported results, although TBOH‐F3 was found to be effective in accelerating the reaction rate and improving the S_N_2:E2 product ratio, further improvements in selectivity are desirable for secondary alkyl bromide substrates. This effect could be achieved by decreasing even more the free energy of the TS(S_N_2)‐ROH transition state (**Figure** **3**). In this work, we modified our strategy to increase hydrogen bonding strength by using bulky diols instead of two CF_3_ groups near the hydroxyl in a mono alcohol because that structure renders the alcohol too acidic.^[^ ^10^ ^]^ Thus, we expanded our investigation aimed at obtaining new insights into how different structures of alcohols change the free energy profile. Using reliable calculations, we investigated two bulky diols, the pinacol (named 2,3‐DIOL) and the fluorinated bulky diol BDMb‐F6 ((S,S) stereoisomer) depicted in Figure 3. For comparison, we have also included TBOH and TBOH‐F3 in this study using a secondary alkyl bromide substrate as a model system, 2‐bromopropane. Our goal is to evaluate the effect of these alcohols on the reaction‐free energy profile and their consequence for the S_N_2:E2 selectivity. Thus, the present study intends to use reliable theoretical methods to identify new potential combinations of 18‐crown‐6 and bulky alcohols able to produce highly selective fluorination of secondary substrates.

## 2 Results and Discussion

### 2.1 Theoretical Free Energy Profile with TBOH and TBOH‐F3

**Figure 4:**
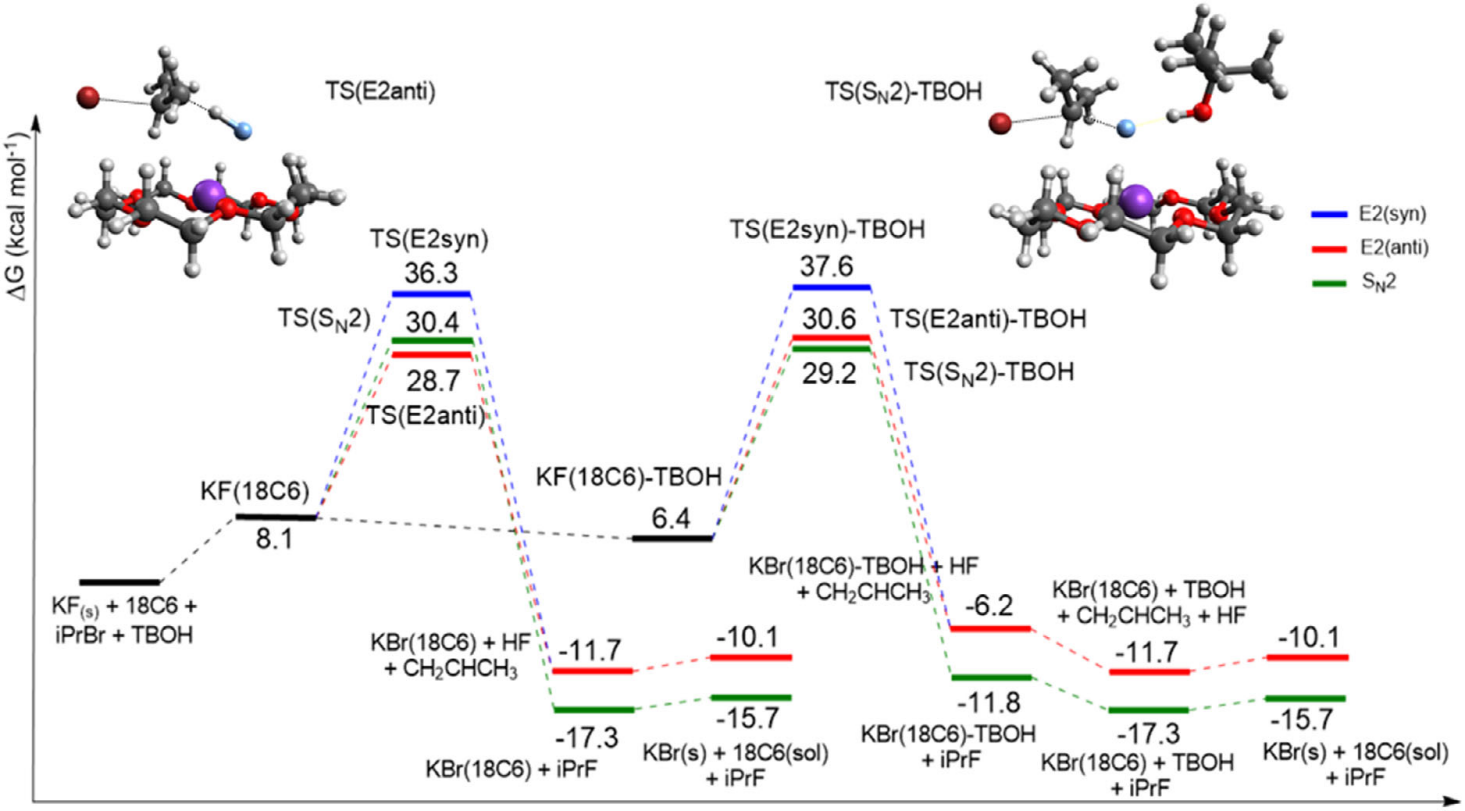
Free energy profile in acetonitrile solution for the reaction KF(18C6) + iPrBr + TBOH. The more stable transition states in each step are shown: TS(E2anti) and TS(S_N_2)‐TBOH, respectively. Calculations using a composite method: ωB97M‐V/ma‐def2‐TZVPP for electronic energy, X3LYP/ma‐def2‐SVP for optimization, frequencies, and conductor‐like polarizable continuum model (CPCM) for solvation free energy. Units of kcal mol^−1^, 1 mol L^−1^ standard state.

The free energy profile shown in **Figure** **4** indicates the solubilization of KF by 18‐crown‐6 ether and the formation of the KF(18C6) complex with Δ*G* = 8.1 kcal mol^−1^. The observable free energy barriers, Δ*G* ^‡^_obs_, for the KF(18C6) complex demonstrate a preference for E2 elimination in the fluorination of 2‐bromopropane, with values of 28.7 and 30.4 kcal mol^−1^ for TS(E2anti) and TS(S_N_2), respectively. As a result, there is a difference of 1.7 kcal mol^−1^ in the effective barriers under anhydrous conditions. Analyzing the selectivity of the reaction at 25 °C, we can calculate the formation of ≈95% of E2 products (k_E2_/(k_E2_ + k_SN2_)–Supporting Information) without the presence of alcohol. In experiments, the presence of water can increase the S_N_2:E2 product ratio, and temperatures close to the reflux of acetonitrile (≈82 °C) can decrease the selectivity. The E2(syn) mechanism has a very high barrier of 36.3 kcal mol^−1^, and it is not expected to be important.

Although the S_N_2 product is thermodynamically more stable than the E2 product, the reaction is kinetically favored for elimination, in agreement with experiments.^[^ ^54^ ^]^ Furthermore, the formation of KBr(18C6) restricts the release of the 18C6 species for promoting a new reaction, and this fact can explain why the crown ether is less efficient kinetically when used in substoichiometric amounts.^[^ ^10^ ^]^ To examine this property clearly, refer to Equation (1), which presents the calculation of the exchange equilibrium considering KBr and KF in the gas phase. Equation 2 presents this same reaction involving the KF_(s)_ and KBr_(s)_ salts, which includes the experimental free energy values for the sublimation. This analysis shows that this exchange equilibrium, the formation of the KF(18C6)_(sol)_ complex from the KBr(18C6)_(sol)_ complex and KF(s), is considerably unfavorable by 9.7 kcal mol^−1^. Therefore, the formation of the KBr(18C6) complex is a limiting factor in the reaction kinetics, requiring stoichiometric or over‐stoichiometric amounts of 18C6 to promote the reaction quickly.

(1) $${\text{KF}}_{\left(g\right)}\text{ + KBr}{\left(\text{18C6}\right)}_{\left(\text{sol}\right)}\;⇌{\text{ KBr}}_{\left(g\right)}\text{ + KF}{\left(\text{18C6}\right)}_{\left(\text{sol}\right)}{\text{ ΔG}}^{*}\text{ = 3}\text{.4 kcal/mol}$$

(2) $${\text{KF}}_{\text{(s)}}\text{ + KBr}{\left(\text{18C6}\right)}_{\left(\text{sol}\right)}⇌{\text{ KBr}}_{\text{(s)}}\text{ + KF}{\left(\text{18C6}\right)}_{\left(\text{sol}\right)}{\text{ ΔG}}^{*}\text{ = 9}\text{.7 kcal/mol}$$

The inclusion of microsolvation by TBOH, as shown in Figure 4, is responsible for a 1.7 kcal mol^−1^ stabilization of the KF(18C6) complex, according to the employed model. This property increases the concentration of the active KF species in acetonitrile and changes the observed free energy of activation, Δ*G* ^‡^_obs_. An interesting effect of the microsolvation by TBOH is the inversion of chemoselectivity compared to the barriers without alcohol, with a 1.4 kcal mol^−1^ preference for S_N_2 over E2. However, the overall barrier for S_N_2 via TS(S_N_2)‐TBOH is ΔG^‡^_obs_ = 29.2 kcal mol^−1^, while the TS(E2(anti)) pathway is only 28.7 kcal mol^−1^. A kinetic analysis of the free energy profile indicates that the S_N_2:E2 product ratio must be dependent on the concentration of TBOH because this property determines the concentration of KF(18C6) and KF(18C6)TBOH species.^[^ ^45^ ^]^ An exact prediction of the selectivity based on Figure 4 requires an analysis of the species in equilibrium, as done for the TBAF(TBOH)_n_ complex.^[^ ^45^ ^]^ Therefore, our findings suggest that incorporating a stoichiometric quantity of TBOH is insufficient to achieve the inversion of chemoselectivity.

**Figure 5:**
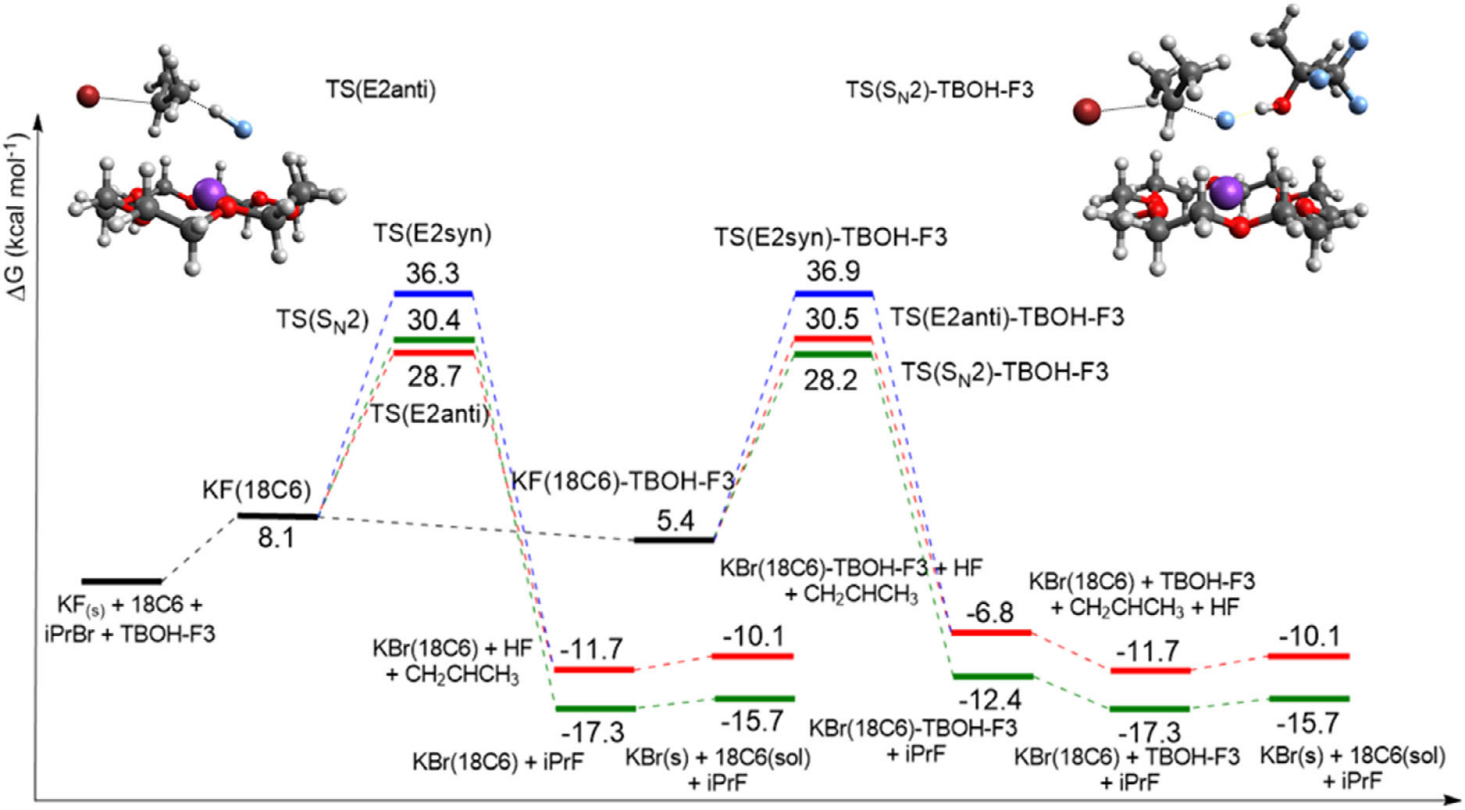
Free energy profile in acetonitrile solution for the reaction KF(18C6) + iPrBr + TBOH‐F3. The more stable transition states in each step are shown: TS(E2anti) and TS(S_N_2)‐TBOH‐F3, respectively. Calculations using a composite method: ωB97M‐V/ma‐def2‐TZVPP for electronic energy, X3LYP/ma‐def2‐SVP for optimization, frequencies, and CPCM for solvation free energy. Units of kcal mol^−1^, 1 mol L^−1^ standard state.

With the introduction of TBOH‐F3 in the reaction with 2‐bromopropane (**Figure** **5**), the solubilization of KF is further increased, and the KF(18C6)‐TBOH‐F3 species has a free energy of 5.4 kcal mol^−1^. We can also observe a reduction of the barrier for the S_N_2 pathway, with Δ*G* ^‡^_obs_ = 28.2 kcal mol^−1^ via TS(S_N_2)‐TBOH‐F3, compared to 30.4 kcal mol^−1^ for the reaction without alcohol. On the other hand, the reaction via the E2(anti) mechanism (TS(E2anti)‐TBOH‐F3) has an increase in the barrier to 30.5 kcal mol^−1^. Thus, this alcohol further enhances the stabilization of the S_N_2 pathway about E2 when compared to the TBOH alcohol (ΔΔ*G* ^‡^ of 2.3 kcal mol^−1^ for TBOH‐F3 versus 1.4 kcal mol^−1^ for TBOH). Considering the overall S_N_2:E2 selectivity, the free energy profile shows that the pathway via TS(S_N_2)‐TBOH‐F3 is the most favorable, staying 0.5 kcal mol^−1^ below the pathway via TS(E2anti). However, the selectivity is dependent on the concentration of alcohol, an experimentally observed fact for a primary substrate.^[^ ^10^ ^]^ In addition, the experiments^[^ ^10^ ^]^ also show that TBOH‐F3 induces more S_N_2:E2 selectivity than TBOH, in agreement with the present calculations. Nevertheless, although TBOH‐F3 is notably superior to TBOH, the fluorination of a secondary substrate using the TBOH‐F3 alcohol still exhibits the considerable formation of E2 products (56%, see Ref. [10]), and further improvement must be made.

### 2.2 Theoretical Free Energy Profile with 2,3‐DIOL and BDMb‐F6

The preceding findings indicate that an increase in the number or strength of hydrogen bonds is required to achieve higher S_N_2:E2 selectivity. More fluorinated bulky alcohols, such as TBOH‐F6, have the drawback of higher acidity and formation of ether side products.^[^ ^10^ ^]^ An alternative is increasing the number of hydroxyl groups. Thus, we have investigated two bulk diols: 2,3‐DIOL and BDMb‐F6. The 2,3‐DIOL utilizes two hydrogen bonds to interact with the fluoride ion, whereas the bulky diol BDMb‐F6 adheres to the transition state stabilization concept introduced by Pliego.^[^ ^70^ ^]^

**Figure 6:**
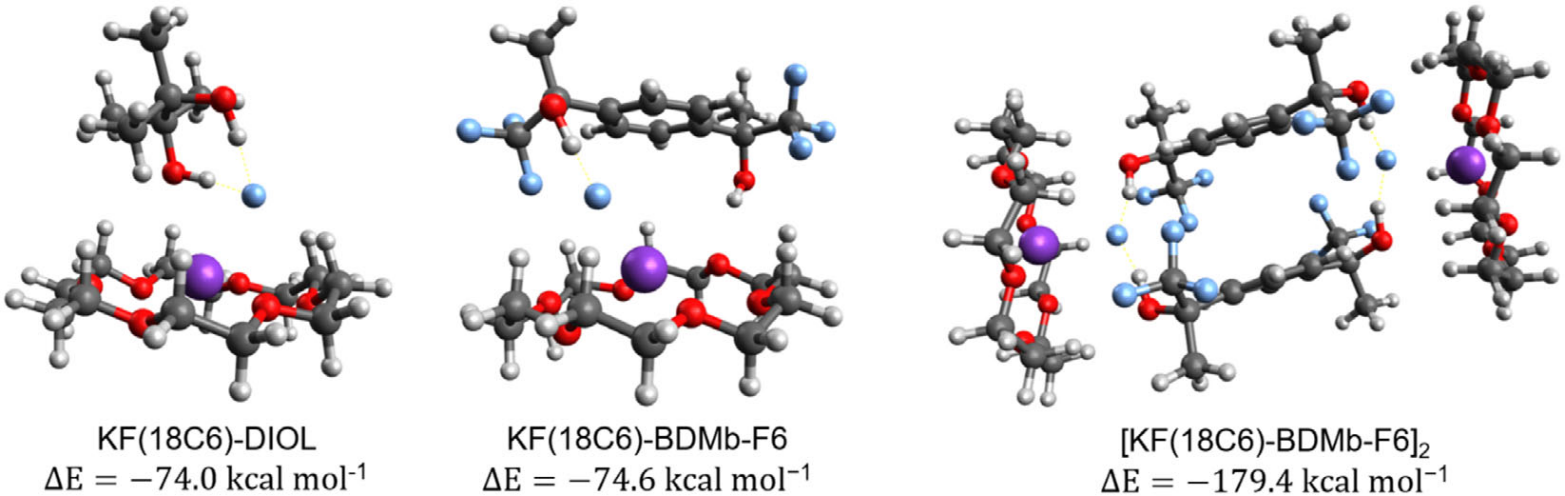
From left to right: optimized geometries in acetonitrile solution of the KF(18C6)‐DIOL, KF(18C6)‐BDMb‐F6 complexes, and the [KF(18C6)‐BDMb‐F6]_2_ dimer. Interaction energies were calculated from the separated species with KF in the gas phase using the ωB97M‐V/ma‐def2‐TZVPP level of theory for electronic energy.

**Figure** **6** displays, sequentially from left to right, the microsolvated complexes formed by 2,3‐DIOL, BDMb‐F6, and the dimer [KF(18C6)‐BDMb‐F6]_2_. The interaction energies (ΔE) are calculated using the ωB97M‐V/ma‐def2‐TZVPP method from the separated species (KF + 18C6 + diol) during complex formation. Considering only KF + 18C6, the interaction energy is −39.9 kcal mol^−1^, meaning that the addition of the 2,3‐DIOL and BDMb‐F6 alcohols should be able to produce a considerable increase in the KF salt solubilization. While 2,3‐DIOL offers two hydrogen bonds by hydroxyl groups with F^−^ and stabilization via interaction between the oxygen of the DIOL and the K^+^ cation, the hydroxyl groups in BDMb‐F6 result in one direct hydrogen bond with the F^−^ anion, and another with the oxygen of the crown ether. The slightly enhanced stabilization of the interaction energy of the KF(18C6)‐BDMb‐F6 complex comes from the greater proton acidity of the —OH groups of the diol, caused by the ‐CF_3_ groups.^[^ ^10^ ^]^ The dimer [KF(18C6)‐BDMbF_6_]_2_ has a substantial stabilization energy, which may be detrimental to the catalytic effect.

**Figure 7:**
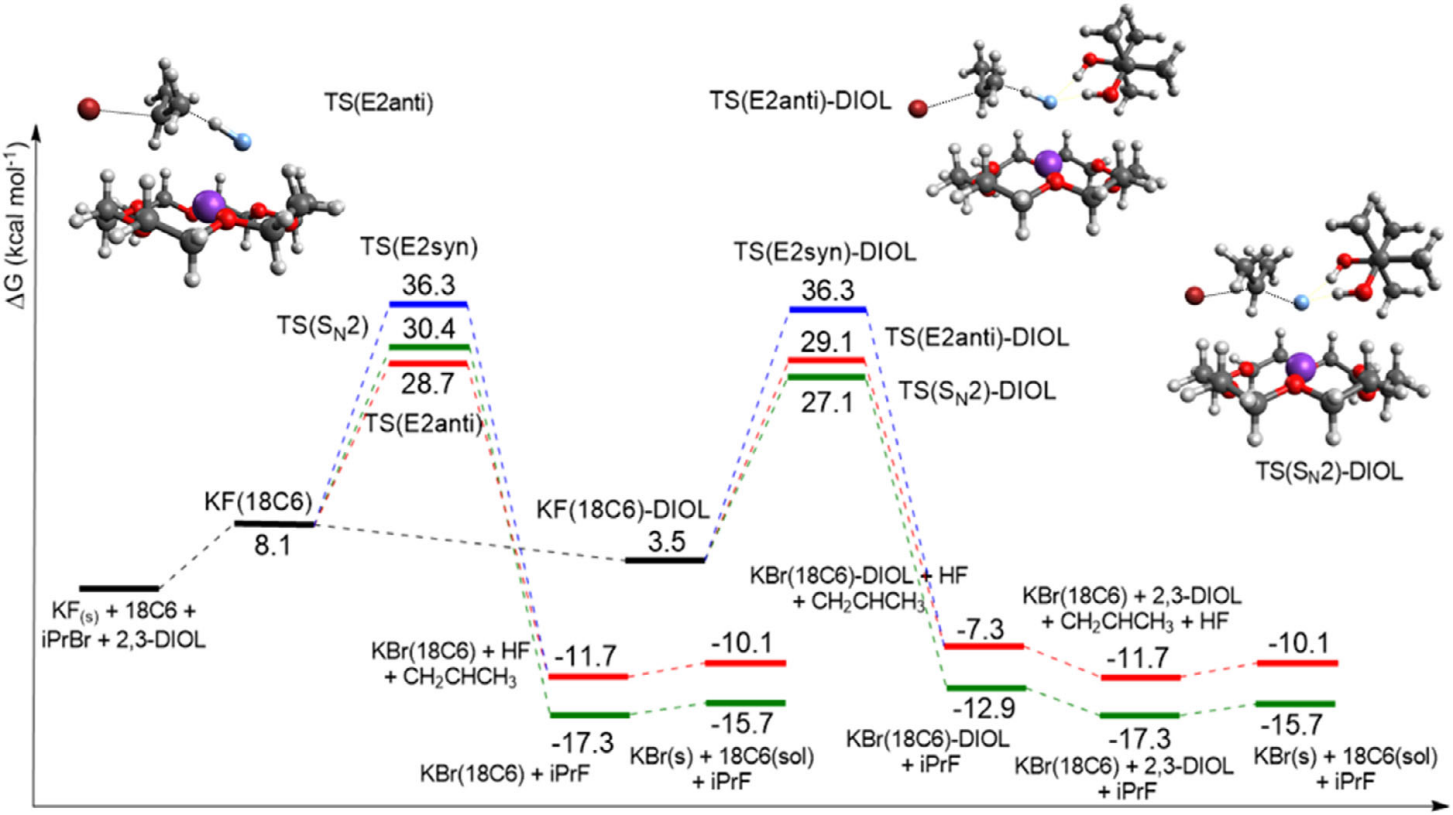
Free energy profile in acetonitrile solution for the reaction KF(18C6) + iPrBr + 2,3‐DIOL. The more important transition states in each step are shown: TS(E2anti), TS(S_N_2)‐DIOL, and TS(E2anti)‐DIOL. Calculations using a composite method: ωB97M‐V/ma‐def2‐TZVPP for electronic energy, X3LYP/ma‐def2‐SVP for optimization, frequencies, and CPCM for solvation free energy. Units of kcal mol^−1^, 1 mol L^−1^ standard state.

**Figure** **7** represents the free energy profile for 2,3‐DIOL. The introduction of this diol leads to greater stabilization of the KF(18C6) complex compared to the other alcohols reported here, with a free energy of 3.5 kcal mol^−1^ for the KF(18C6)‐DIOL complex. The barrier via TS(S_N_2)‐DIOL is further decreased, with a value of Δ*G* ^‡^_obs_ = 27.1 kcal mol^−1^. Unexpectedly, the barrier via E2 is also stabilized for this diol, with Δ*G* ^‡^_obs_ = 29.1 kcal mol^−1^. Thus, the S_N_2 barrier is 2.0 kcal mol^−1^ lower than the E2 barrier with the presence of the 2,3‐DIOL, while the TBOH‐F3 alcohol has a better selectivity with a difference of 2.3 kcal mol^−1^. On the other hand, the ΔG^‡^_obs_ via TS(S_N_2)‐DIOL is 1.6 kcal mol^−1^ lower than the competitive TS(E2anti) pathway, compared with a difference of only 0.5 kcal mol^−1^ calculated for the TBOH‐F3. Consequently, although the SN2:E2 selectivity depends on the 2,3‐DIOL concentration, the present calculations indicate that this diol should outperform the TBOH‐F3 alcohol in selectivity. Furthermore, it should also be kinetically more effective.

**Figure 8:**
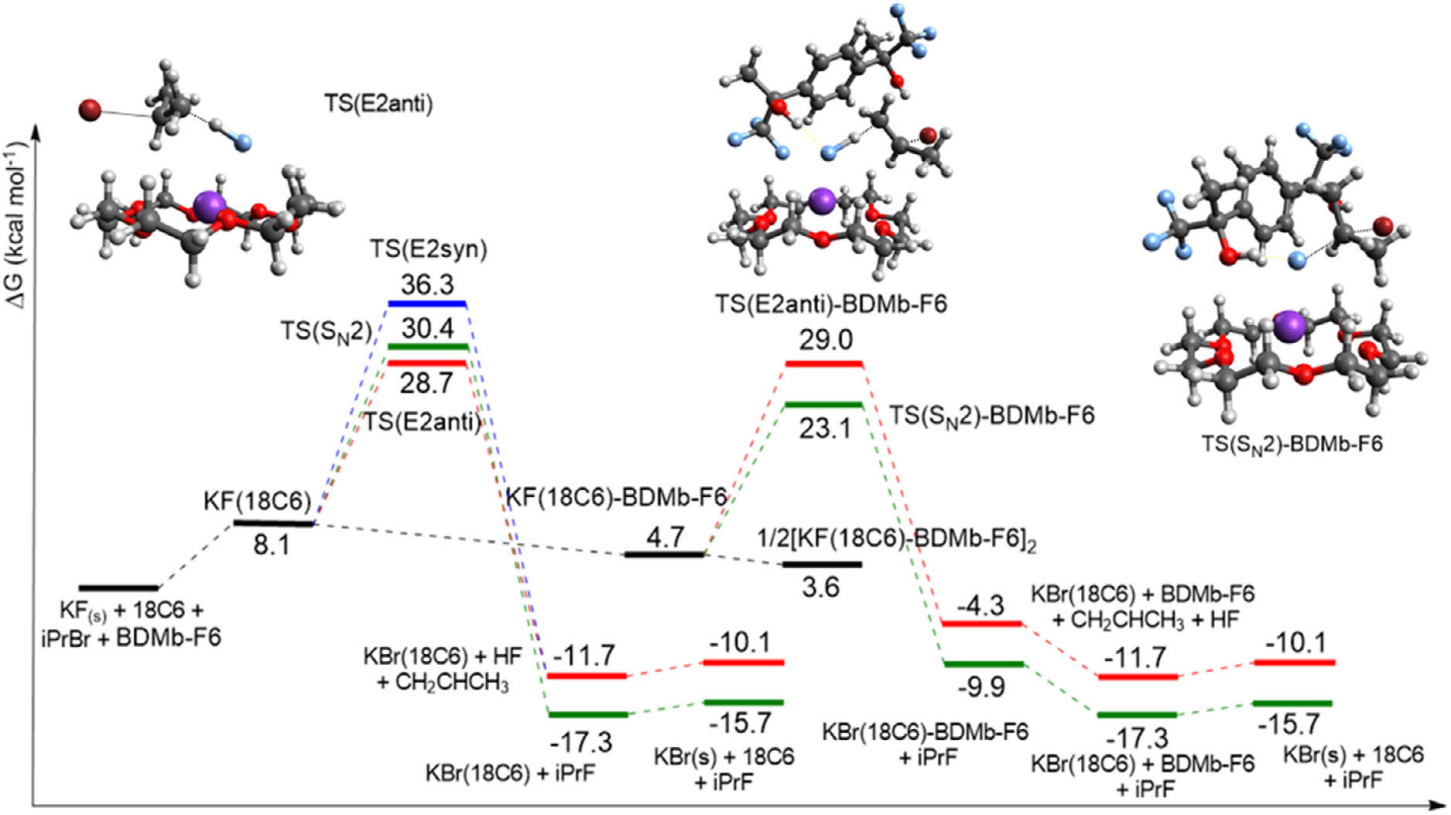
Free energy profile in acetonitrile solution for the reaction KF(18C6) + iPrBr + BDMb‐F6. The more important transition states in each step are shown: TS(E2anti), TS(S_N_2)‐BDMb‐F6, and TS(E2anti)‐BDMb‐F6. Calculations using a composite method: ωB97M‐V/ma‐def2‐TZVPP for electronic energy, X3LYP/ma‐def2‐SVP for optimization, frequencies, and CPCM for solvation free energy. Units of kcal mol^−1^, 1 mol L^−1^ standard state.

Regarding the BDMb‐F6 diol (**Figure** **8**), it offers less stabilization when added to the KF(18C6) complex compared to 2,3‐DIOL, presenting a free energy of 4.7 kcal mol^−1^. However, it forms a more stable species, the [KF(18C6)‐BDMb‐F6]_2_ dimer, with a free energy of 3.6 kcal mol^−1^, enhancing the solubilization of KF and being as effective as 2,3‐DIOL. However, it is important to consider that modeling such a complex system is not straightforward, and the Grimme method^[^ ^71^ ^]^ used for the calculation of the vibrational contribution to the free energy should lead to a less stable dimer. Therefore, we believe that this species is even more stable than presented in Figure 8, increasing the solubilization of KF.

The most interesting effect of BDMb‐F6 is on the stabilization of competitive transition states. Indeed, the BDMb‐F6 alcohol produces very high stabilization for the S_N_2 transition state by two hydrogen bonds to the incoming fluoride ion and leaving the bromide ion (Figure 8). The additional hydrogen bonding to the leaving Br^−^ group has a considerable effect on reducing the free energy barriers in the solution phase, particularly for the S_N_2 transition state. The effect on the free energy profile is noteworthy, producing a substantially lower barrier via TS(S_N_2)‐BDMb‐F6, with Δ*G* ^‡^_obs_ = 23.1 kcal mol^−1^. For comparison, the barrier via TS(E2anti)‐BDMb‐F6 corresponds to ΔG^‡^_obs_ = 29.0 kcal mol^−1^. This significant stabilization of S_N_2 relative to E2 (5.9 kcal mol^−1^) is in line with the idea of S_N_2 transition state stabilization via multiple hydrogen bonds.^[^ ^70^ ^]^ On the other hand, the reaction via TS(E2anti)‐BDMb‐F6 is slightly less favorable (0.3 kcal mol^−1^) than the reaction via TS(E2anti), supporting the view that a microenvironment with this kind of structure is very effective for selective fluorination.

Analyzing all the pathways in Figure 8, we can see that the reaction via TS(S_N_2)‐BDMb‐F6 is 5.6 kcal mol^−1^ below the pathway via TS(E2anti), indicating this alcohol should induce a very high chemoselectivity when using a reasonable concentration of BDMb‐F6. Its concentration should be greater than that of 18‐crown‐6 to inhibit the reaction via KF‐18C6. An interesting observation is that the formation of stable aggregates could decrease the overall reaction rate. However, if all the crown ether forms the complex, a very high S_N_2:E2 selectivity should be observed in secondary substrates in the absence of other hidden pathways leading to elimination. In summary, this work identified the BDMb‐F6 compound as a highly promising diol for use with 18‐crown‐6 in the selective fluorination process using KF. This compound is not readily available, which underscores the significance of theoretical calculations to identify potential catalysts or mediators for inducing selective fluorination before conducting synthesis and testing.

## 3 Conclusion

The effect of combining 18C6 with the bulky alcohols TBOH, TBOH‐F3, 2,3‐DIOL (pinacol), and BDMb‐F6 in the nucleophilic fluorination of secondary substrates with KF salt was studied theoretically using reliable calculations. Our results point out that in the absence of these alcohols, the E2 side product is the main one, while introducing these alcohols improved the S_N_2:E2 selectivity. The effect of these alcohols to induce more S_N_2 selectivity is in the order: TBOH < TBOH‐F3 < 2,3‐DIOL < BDMb‐F6. Notably, we have identified BDMb‐F6 as a highly effective bulky diol, capable of inducing significant selectivity and rate acceleration effects.

## 4 Theoretical Methods

The mechanisms of fluorination reactions in the condensed phase (acetonitrile) were investigated using density functional theory. Free energy profiles were calculated by first obtaining the geometries of stationary points, which were optimized using the X3LYP functional^[^ ^72^ ^]^ with the def2‐SVP basis set.^[^ ^73^ ^]^ This function has a slightly better performance than B3LYP for properties such as heats of formation, ionization potentials, electron affinities, and total atomic energies. In addition, it outperforms B3LYP for hydrogen‐bonded complexes.^[^ ^74^ ^]^ The ma‐def2‐SVP basis set, augmented with diffuse s and p functions, was employed for F, O, and Br atoms.^[^ ^75^ ^]^ For the solvation free energy (ΔG_solv_), it was utilized the CPCM model^[^ ^76^, ^77^ ^]^ with solvent excluded surface type cavities,^[^ ^78^ ^]^ with a solvent radius of 2.49 Å.^[^ ^79^ ^]^ The nonelectrostatic component of ΔG_solv_ was calculated using a surface area‐dependent term^[^ ^79^ ^]^ with the GEPOL (algorithm) for the construction of atomic cavities.^[^ ^80^ ^]^ Additionally, a scaling factor of 1.35 was applied to all atoms with the following radii: H (1.10 Å), C (1.70 Å), O (1.52 Å), F (1.28 Å), and Br (1.85 Å).^[^ ^79^ ^]^ To obtain more accurate electronic energy values, the reliable ωB97M‐V functional^[^ ^81^ ^]^ and def2‐TZVPP basis set were employed, with the ma‐def2‐TZVPP basis set used for F, O, and Br atoms. The ωB97M‐V functional is among the best‐performing functionals for chemical reactions.^[^ ^82^ ^]^

The thermodynamic properties of the condensed‐phase species were determined by analytical harmonic frequency calculations at the same level of theory used for geometry optimization. All calculations were performed using the ORCA 5.0.3 program.^[^ ^83^, ^84^ ^]^ The molecular contribution to the Gibbs free energy (G^*^_mol_) is given by Equation 3 in the standard state of 1 mol/L, where E_ele_ is the electronic energy and G^*^_vrt_ corresponds to the vibrational, rotational, and translational contributions to the free energy. The Grimme approximation was used for low frequencies,^[^ ^71^ ^]^ as implemented in ORCA. The chemical potential in the condensed phase for each species (G^*^_sol_) is given by Equation 4, where ΔG^*^_solv_ is the solvation free energy. Equation 5 provides the rate constant, k(T) (see Supporting Information), in the condensed phase through the free energy of activation (ΔG^‡^), according to the Eyring transition state theory, where *k*_*B*_ is the Boltzmann constant, *h* is the Planck constant, *T* is the absolute temperature (298.15 K), and *R* is the ideal gas constant.

(3) $$G_{mol}^{*}=E_{ele}+G_{vrt}^{*}$$

(4) $$G_{sol}^{*}=G_{mol}^{*}+\Delta G_{solv}^{*}$$

(5) $$k\left(T\right)=\frac{k_{B}T}{h}e^{-\frac{\Delta G_{sol}^{‡}}{RT}}$$

Regarding the solubilization of the KF salt, this property will influence the observable free energy barrier (ΔG^‡^_obs_) and can be understood in three steps. 1) Contribution of the sublimation free energy of the salt (ΔG_1_), forming the gaseous phase ion pair, whose value for KF is 48.29 kcal mol^−1^ and for KBr is 41.95 kcal mol^−1^, at the standard state of 1 mol L^−1^ according to the NIST database,^[^ ^85^ ^]^ Equation (6). 2) free energy for the formation of the complex with the 18‐crown‐6 countercation in solution (ΔG_2_), Equation (7). The sum of these two steps is equivalent to the solubilization free energy of KF with the crown ether (ΔG_solub_ = ΔG_1_ + ΔG_2_). 3) Equation (8) describes the activation process for the formation of the transition state between the KF(18C6) complex and the substrate, ΔG^‡^_3_. Therefore, ΔG^‡^_obs_ can be obtained through Equation 9 for cases where ΔG_solub_ > 0.

(6) $${\text{KF}}_{\left(s\right)}⇌{\text{KF}}_{\left(g\right)}\;\;\;\;\;\;{\text{ΔG}}_{1}\text{=48}\text{.29 kcal/mol}$$

(7) $${\text{KF}}_{\left(g\right)}{\text{ + 18C6}}_{\left(sol\right)}⇌\text{ KF}{\left(\text{18C6}\right)}_{\left(sol\right)}{\text{ ΔG}}_{2}$$

(8)

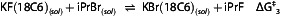



(9)

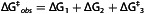




## Conflict of Interest

The authors declare no conflict of interest.

## Supporting information

Supplementary Material

## Data Availability

The data that support the findings of this study are available from the corresponding author upon reasonable request.
