# Supplementary material for: Nucleophilic Fluorination of a Secondary Alkyl Bromide with KF(18‐Crown‐6) and Bulky Diols: Microsolvation Causes Chemoselectivity Inversion in the Free Energy Profile
Source: Chempluschem. 2025 Jul 1;90(8):e202500257. doi: 10.1002/cplu.202500257 (PMC12352739; doi:10.1002/cplu.202500257)
Supplement: Supplementary file 1 — Supplementary Material [file CPLU-90-e202500257-s001.pdf]

Derivation of the rate law

In the present study, from the free energy profile (Figure 6) we can write that the concentration of the KF-18C6-BDMbF$_6$ complex in equilibrium is:

$$[\text{KF(18C6)-BDMb-F6}] = K_1.[\text{18C6}].[\text{BDMb-F6}]$$

$$K_1 = e^{-\Delta G_1/RT}$$

Observe that KF is a solid and does not enter in the equation. The activation step is via TS(S$_N$2)-BDMb-F6 from KF(18C6)-BDMb-F6, with a barrier $\Delta G^{\ddagger} = 23.1 - 4.7 = 18.4$ kcal mol^{-1}. By transition state theory, we can write:

$$rate = k_2[\text{KF(18C6)-BDMb-F6}].[\text{RBr}]$$

With

$$k_2 = \frac{k_b T}{h} e^{-\Delta G_2^{\ddagger}/RT}$$

Combining the equations, the final rate law becomes:

$$rate = K_1 k_2[\text{18C6}].[\text{BDMb-F6}].[\text{RBr}]$$

$$K_1 k_2 = \frac{k_b T}{h} e^{-(\Delta G_1 + \Delta G_2^{\ddagger})/RT}$$

and the effective barrier is $\Delta G_1 + \Delta G^{\ddagger}_2$ = 23.1 kcal mol^{-1}

Kinetic and Thermodynamic data for the reactions

Table 1: Kinetic data for KF reaction with 2-bromopropane catalyzed by 18-crown-6, TBOH, TBOH-F3, 2,3-DIOL, and BDMb-F6.

Kinetics ROH	$\Delta E_{ele}^{\ddagger *}$ [b]	$\Delta G_{g}^{\ddagger *}$ [c]	$\Delta\Delta G_{solv}^{\ddagger *}$ [d]	$\Delta G_{sol}^{\ddagger *}$ [e]	k_{eff} [f]
$KF_{(s)} \rightarrow KF_{(g)}$		48.29			
$KBr_{(s)} \rightarrow KBr_{(g)}$		41.95			
$KF(18C6) + iPrBr \rightarrow TS(S_N2)$	1.72	12.41	9.96	22.36	$3.2\ 10^{-10}$
$KF(18C6) + iPrBr \rightarrow TS(E2anti)$	5.26	13.65	6.96	20.61	$5.7\ 10^{-9}$
$KF(18C6) + iPrBr \rightarrow TS(E2syn)$	5.69	14.10	14.08	28.17	$1.5\ 10^{-14}$
$KF(18C6)\text{-}TBOH + iPrBr \rightarrow TS(S_N2)\text{-}TBOH$	9.65	20.83	1.93	22.76	$6{,}4\ 10^{-10}$
$KF(18C6)\text{-}TBOH + iPrBr \rightarrow TS(E2anti)\text{-}TBOH$	18.92	26.84	-2.65	24.19	$3{,}6\ 10^{-14}$
$KF(18C6) - TBOH + iPrBr \rightarrow TS(E2syn)\text{-}TBOH$	16.70	24.77	6.45	31.22	$1{,}9\ 10^{-16}$
$KF(18C6)\text{-}TBOH\text{-}F3 + iPrBr \rightarrow TS(S_N2)\text{-}TBOH\text{-}F3$	11.29	22.82	~0.00	22.82	$1.3\ 10^{-8}$
$KF(18C6)\text{-}TBOH\text{-}F3 + iPrBr \rightarrow TS(E2anti)\text{-}TBOH\text{-}F3$	19.90	29.11	-3.97	25.14	$5.7\ 10^{-9}$
$KF(18C6)\text{-}TBOH\text{-}F3 + iPrBr \rightarrow TS(E2syn)\text{-}TBOH\text{-}F3$	17.22	26.28	5.25	31.52	$5.6\ 10^{-15}$
$KF(18C6)\text{-}DIOL + iPrBr \rightarrow TS(S_N2)\text{-}DIOL$	11.36	23.29	0.28	23.57	$8.5\ 10^{-8}$
$KF(18C6)\text{-}DIOL + iPrBr \rightarrow TS(E2anti)\text{-}DIOL$	23.18	30.61	-5.03	25.58	$2.9\ 10^{-9}$
$KF(18C6)\text{-}DIOL + iPrBr \rightarrow TS(E2syn)\text{-}DIOL$	20.99	28.41	4.43	32.84	$1.5\ 10^{-14}$
$KF(18C6)\text{-}BDMb\text{-}F6 + iPrBr \rightarrow TS(S_N2)\text{-}BDMb\text{-}F6$	4.66	16.13	2.28	18.41	$7.0\ 10^{-5}$
$KF(18C6)\text{-}BDMb\text{-}F6 + iPrBr \rightarrow TS(E2anti)\text{-}BDMb\text{-}F6$	17.56	25.45	-1.12	24.33	$3.3\ 10^{-9}$

a) Standard state of 1 mol L^{-1}, temperature of 298.15 K and units in kcal mol^{-1}; b) ωB97M-V/ma-def2-TZVPP for electronic energy; c) Molecular contribution to the free energy at X3LYP/ma-def2-SVP. This value is close to the gas phase free energy; d) Variation of the solvation free energy – solvent effect using CPCM model; e) Condensed phase free energy barriers for the acetonitrile solvent; f) kinetic constants in s^{-1} and 298.15 K. This value includes the observable free energy barriers of free energy profiles.

Table 2: Thermodynamics data for KF reaction with 2-bromopropane catalyzed by 18-crown-6, TBOH, TBOH-F3, 2,3-DIOL, and BDMb-F6.

**Thermodynamics ROH**	ΔE^{*}_{ele} [b]	$\Delta G^{*}_{gás}$ [c]	$\Delta\Delta G^{*}_{solv}$ [d]	ΔG^{*}_{sol} [e]
$KF_{(g)} + 18C6_{(sol)} \rightarrow KF(18C6)_{(sol)}$	-39.85	-28.89	-11.32	-40.21
$KF(18C6) + iPrBr \rightarrow KBr(18C6) + iPrF$ **(S_N2)**	-27.34	-25.96	0.58	-25.38
$KF(18C6) + iPrBr \rightarrow KBr(18C6) + CH_3CHCH_2 + HF$ **(E2)**	-7.30	-17.31	-2.48	-19.78
$KBr(18C6)_{(sol)} \rightarrow KBr_{(g)} + 18C6_{(sol)}$	44.86	33.06	10.50	43.56
$KF(18C6) + TBOH \rightarrow KF(18C6) - TBOH$	-26.80	-14.77	13.11	-1.66
$KF(18C6) + TBOH\text{-}F3 \rightarrow KF(18C6) - TBOH\text{-}F3$	-30.60	-18.54	15.81	-2.73
$KF(18C6) + DIOL \rightarrow KF(18C6) - DIOL$	-34.51	-19.81	15.23	-4.59
$KF_{(g)} + (18C6) + DIOL \rightarrow KF(18C6) - DIOL$	-74.37	-48.70	3.91	-44.79
$KF(18C6) + BDMb\text{-}F6 \rightarrow KF(18C6) - BDMb\text{-}F6$	-34.79	-19.10	15.72	-3.38
$2KF(18C6) - BDF6 \rightarrow [KF(18C6) - BDMb\text{-}F6]$	-30.19	-18,19	15.96	-2.24
$2KF_{(g)} + 2(18C6) + 2BDMb\text{-}F6 \rightarrow [KF(18C6) - BDMb\text{-}F6]$	-179.48	-114.18	24.77	-89.41
$KF(18C6)\text{-}TBOH + iPrBr \rightarrow iPrF + KBr(18C6)\text{-}TBOH$ **(S_N2)**	-17.10	-15.95	-2.28	-18.23
$KF(18C6)\text{-}TBOH + iPrBr \rightarrow KBr(18C6)\text{-}TBOH + iPr + HF$ **(E2)**	2.94	-7.29	-5.34	-12.63
$KBr(18C6)\text{-}TBOH \rightarrow KBr(18C6) + TBOH$	17.06	6.26	-11.70	-5.44
$KF(18C6)\text{-}TBOH\text{-}F3 + iPrBr \rightarrow iPrF + KBr(18C6)\text{-}TBOH\text{-}F3$ **(S_N2)**	-14.45	-13.20	-4.59	-17.79
$KF(18C6)\text{-}TBOH\text{-}F3 + iPrBr \rightarrow KBr(18C6)\text{-}TBOH\text{-}F3 + CH_3CHCH_3 + HF$ **(E2)**	5.66	-4.47	-7.65	-12.12
$KBr(18C6)\text{-}TBOH\text{-}F3 \rightarrow KBr(18C6) + TBOH\text{-}F3$	19.23	7.08	-11.98	-4.90
$KF(18C6)\text{-}DIOL + iPrBr \rightarrow iPrF + KBr(18C6)\text{-}DIOL$ **(S_N2)**	-12.57	-12.52	-3.84	-16.37
$KF(18C6)\text{-}DIOL + iPrBr \rightarrow KBr(18C6)\text{-}DIOL + CH_3CHCH_3 + HF$ **(E2)**	7.47	-3.86	-6.90	-10.76
$KBr(18C6)\text{-}DIOL \rightarrow KBr(18C6) + DIOL$	19.74	6.37	-10.80	-4.43
$KF(18C6)\text{-}BDMb\text{-}F6 + iPrBr \rightarrow iPrF + KBr(18C6)\text{-}BDMb\text{-}F6$ **(S_N2)**	-10.71	-10.15	-4.49	-14.64
$KF(18C6)\text{-}BDMb\text{-}F6 + iPrBr \rightarrow KBr(18C6)\text{-}BDMb\text{-}F6 + CH_3CHCH_3 + HF$ **(E2)**	9.33	-1.49	-7.55	-9.04
$KBr(18C6)\text{-}BDMb\text{-}F6 \rightarrow KBr(18C6) + BDMb\text{-}F6$	18.16	3.29	-10.65	-7.36

a) Standard state of 1 mol L^{-1}, temperature of 298.15 K and units in kcal mol^{-1}; b) ωB97M-V/ma-def2-TZVPP for electronic energy; c) Molecular contribution to the free energy at X3LYP/ma-def2-SVP. This value is close to the gas phase free energy; d) Variation of the solvation free energy – solvent effect using CPCM model; e) Condensed phase free energy barriers for the acetonitrile solvent.

Coordinates of the optimized structures

Structures with additional low vibrational frequencies were included in the calculation of the free energy considering a frequency of 10 cm^{-1} and the Grimme treatment of low vibrational modes.

TBOH

C	0.58479360445812	-0.06043045036718	0.30096977974201
C	0.68779884306986	-1.43524035513216	-0.37305082894243
H	1.30148880128178	-2.11620114773250	0.23611606585780
H	1.14082204919495	-1.35840990770333	-1.37425237487001
H	-0.31095517192512	-1.88853435169527	-0.49417604439652
C	-0.29825158085515	0.88510898746219	-0.52413010475305
H	-0.38967416630935	1.86011344954254	-0.02169392552239
H	-1.31166869971652	0.46671886998482	-0.64895096880130
H	0.12023780387338	1.04855459690813	-1.52991968902933
C	1.97184717846047	0.54394349098739	0.52026476488332
H	2.48723148637957	0.70229631482575	-0.43951963069368
H	2.58607663920777	-0.12460957649549	1.14230433604448
H	1.88899715583324	1.51185423196163	1.03742269621039
O	0.02790891605103	-0.21124469176176	1.61599390466410
H	-0.85665285900403	-0.59156946078479	1.54162201960662

TBOH-F3

C	0.75349733165225	0.15870959584952	0.11550673806908
C	0.72271690528340	-1.28195358003971	-0.40800999228545
H	1.12659959826332	-1.94592973399080	0.36946913476155
H	1.32842791354997	-1.39421571218483	-1.31831289306449
H	-0.30559417168426	-1.60094273328072	-0.63924312142322
C	0.13247780913134	1.10726746965194	-0.93966340900556
C	2.17295732815402	0.61517563985777	0.44589219660174
H	2.80992185554274	0.60585131510695	-0.44992345256916
H	2.60022072096628	-0.06795149092645	1.19336566718935
H	2.16085032406815	1.62909406531671	0.86812722703501
O	-0.00474781677768	0.28721092246267	1.30589557316957
H	-0.90457981642259	-0.03308310656394	1.15554453180260
F	0.19243719088756	2.39390309777365	-0.57370731467535
F	0.74555650506761	1.00036711435267	-2.13437301397323
F	-1.17419167768206	0.81284713661455	-1.13600787163243

2,3-DIOL

C	-2.31675092792289	0.22263453736328	-0.58486160511465
C	-0.93671659158057	0.65930169621313	0.04828447108830
C	-0.28635561368179	1.79561908732311	-0.75051140516666
H	0.08312047387773	1.45004024132779	-1.72686408299677
H	0.57296042657482	2.18943141155717	-0.18644764269146
H	-1.00183693426692	2.61403542477945	-0.92725479174375
C	0.04307583094676	-0.50456672358063	0.20940589968996
H	0.22091491807700	-1.02065412120698	-0.74575250884141
H	-0.32770357703563	-1.23538919337131	0.94118802285933
H	1.00720013546562	-0.12452585444188	0.57905811114651
O	-3.22779503516017	1.32459176229644	-0.43989585783013
C	-2.90243243912405	-1.00104322410805	0.12992264824710
H	-2.91791395696431	-0.85166484855863	1.22098374487643
H	-2.32404133759070	-1.91243386981979	-0.07979497474073
H	-3.93221522401860	-1.16829715324113	-0.22117989696811

O -1.19867421034645 1.12243282643305 1.38332838767778
C -2.22869036458316 -0.03437199172427 -2.09045732738629
H -2.00314121381444 0.89152285023876 -2.63704719422850
H -3.19607546630407 -0.40917891913375 -2.45704920447551
H -1.45831448263459 -0.78238477670853 -2.32969908860156
H -1.52787581379797 2.02840381636063 1.32796017051561
H -3.57469859611563 1.31140702200214 0.46099412468451

BDMb-F6

F 0.17772436265449 8.12029160338707 -2.29520225762263
H -2.17630489990137 9.05176087096960 -2.65669983620957
C -0.39401743825721 7.56163186768945 -1.22180654206911
C -2.63411940358380 8.55302511973678 -1.79091789696382
O -1.94592274698452 6.46588281597703 -2.65964713001137
F -0.20643591404331 8.41277665216806 -0.19327782331186
H -2.61932049962282 9.23012988153132 -0.92624493522749
F 0.29758676402597 6.43585596393493 -0.94456526681919
H -3.67845315209211 8.31441160641318 -2.03326220827269
C -1.89361745738437 7.24781071629721 -1.47832217618300
H -1.55887401204240 5.59545717212851 -2.49055204818210
C -2.55800825568982 6.50504104917481 -0.30585199221305
H -4.11113671258253 5.74580492548765 -1.60117978959425
H -1.20122462134219 7.07846865228567 1.29302461614184
C -3.73070372661855 5.78419701951930 -0.57853442162087
C -2.10004528652307 6.52904982339004 1.01878065893995
C -4.42132169442575 5.10733172949249 0.42743640884535
C -2.78613485187267 5.84535612513296 2.02347829099431
H -5.32817742309153 4.56760274553631 0.15825033185472
C -3.95381819575746 5.11947657562840 1.75000323614484
H -2.40067319970520 5.87620990065574 3.04400056030094
C -4.66079182918930 4.43815908798836 2.93523531333345
F -4.71980102310843 2.31352089777361 1.83375974078674
H -3.07950841104920 3.39429717259320 3.45845297689942
F -6.53607069684787 3.50466365541453 1.72221620994149
H -6.34809494682054 5.81880602407824 2.99214348473113
H -4.96395686044280 6.26584025126888 4.02689218224456
O -3.71664583282251 3.98215251330995 3.88816084546689
C -5.48704251312815 3.19448822752453 2.50960298694074
C -5.57481618067469 5.42709339739075 3.66670487722355
F -5.97960993959876 2.55073283671415 3.57503978274801
H -6.05206340147741 4.94057311940704 4.52918182076304

Ether 18-Crown-6 (18C6)

O -2.57683666727819 -0.59475522057387 -2.84510113065700
C -3.88086045029376 -0.82586986140797 -2.37528240508938
C -3.88531674073264 -1.97687455219419 -1.39232846626609
O -3.37681502826620 -1.55604049608622 -0.15197081663616
O -1.19053509770243 -1.90415223405398 1.82449705615977
C -0.49770480060981 -1.32290359159521 2.90001356419029
C 0.95124215283039 -1.09553813640483 2.52666690913436
O 1.06401415813567 0.01897438894895 1.67845963554484
C 2.36790918271876 0.25714396648541 1.21226765453059
C 2.36809423425284 1.40553568900544 0.22627631221522
O 1.86451618444388 0.97889570680132 -1.01414229609463

C	1.73335121313037	2.00382878928984	-1.96639193426952
C	1.06147742861811	1.47295564862802	-3.21455035191257
O	-0.31816025602645	1.32280612812483	-2.99450646361694
C	-1.01228110958692	0.74170700419826	-4.06908081710356
C	-2.46167980662259	0.51826704745673	-3.69490351741527
H	-4.92703856946563	-2.34633845342118	-1.27832438111982
H	-3.27902803001154	-2.80815722605789	-1.80385804436829
H	-0.95603185659005	-0.35496922466316	3.18450488509548
H	-0.53747223649867	-1.98730172588584	3.78978424811715
H	1.34796114646794	-2.00560511421528	2.03418563997601
H	1.53848104509670	-0.93445102160489	3.45607295504328
H	3.40801914897761	1.78044679080657	0.11360534994207
H	1.75634116609581	2.23462414702047	0.63414080602819
H	1.52270196707710	0.50434155437284	-3.49195249159277
H	1.24501836149939	2.18282952250676	-4.04942234984254
H	-0.97095368500506	1.40455138280411	-4.95994154844952
H	-0.55652739115564	-0.22777806393531	-4.35255643893916
H	-3.04998706867222	0.35758214753012	-4.62369579983193
H	-2.85549807788068	1.43021616968360	-3.20356925331724
H	-4.55774644773954	-1.07395758632660	-3.22077405902051
H	-4.28525932815612	0.07808384750088	-1.87788951092688
H	1.13365980182766	2.84256558388489	-1.56056149341503
H	2.73163948140159	2.40735392697999	-2.24061082177658
H	3.04048907927826	0.51252913216963	2.05906156912774
H	2.77999078363871	-0.64551791050487	0.71884733175396
C	-2.56985940742893	-2.05795256257778	2.04510069786996
C	-3.24181483362896	-2.58500839466135	0.79545152152150
H	-3.03236595070400	-1.09122701505812	2.32679569449459
H	-2.75107878527910	-2.77173062529975	2.87712831634760
H	-4.23874374890014	-2.99231692575860	1.06899504027418
H	-2.64050116125551	-3.42022263191177	0.38477920429460

2-Bromopropane (iPrBr)

C	-0.98593941965466	2.02814872323699	-0.15823557663063
C	-0.36067664967633	0.77168555560042	0.42146267484731
H	-1.72817332374556	1.78295238860128	-0.93369599823922
H	-0.19516331037025	2.64217570997671	-0.62385220900295
H	-1.47779494462437	2.63398646752019	0.61608246426589
C	0.32930037374046	-0.10741217756689	-0.60667270646133
H	1.14373437051431	0.46607390130812	-1.08296377153278
H	-0.37081860070693	-0.42438384443261	-1.39520590367609
H	0.76643195143367	-1.00565795137738	-0.14783080513543
Br	-1.76898214418855	-0.29534260837472	1.36731413169716
H	0.32863169727823	1.02463383550789	1.23843769986807

KF(18C6)

C	-2.88318065040036	-2.31992337381810	-3.81944104423990
O	-1.69938050470429	-3.06425778454312	-3.64230746451137
C	-3.96099768730452	-2.85601019048652	-2.90524633161551
C	-0.67728695629780	-2.75183546915973	-4.56840078986353
C	0.60348048299478	-3.45280679441183	-4.16872247725984
O	1.00884337359502	-2.95377128101145	-2.92184546005862
O	-3.62294760243732	-2.62722121917750	-1.55346950020446
C	2.34947341135084	-3.20511131450782	-2.54607777312785

C	2.59820322862041	-2.58449687333772	-1.19012756774738
C	-4.54834407507577	-3.18827210790966	-0.64700932749302
C	-4.11289645842519	-2.93831475041782	0.77855088815754
O	-2.91784932108525	-3.63709626111281	1.04031714291809
O	1.91182456944156	-3.34944473197513	-0.20985756053855
C	-2.49276867530998	-3.57042523213694	2.38550669380100
C	1.99994827908137	-2.79783038750103	1.09293974991820
C	1.16081482278263	-3.63560190533547	2.02999388591066
C	-1.11189168689700	-4.17485477083906	2.51070685031633
O	-0.20978254996536	-3.39557476721287	1.76789193959710
H	-3.24378342006177	-2.40206991802966	-4.86468478546364
H	-2.70135813241103	-1.24677315596821	-3.61114190410833
H	-4.91947149589677	-2.35567408072381	-3.15102343892882
H	-4.08847872882161	-3.94105809110252	-3.08638384608342
H	-0.49963940107739	-1.65951161572678	-4.58634743448804
H	-0.97239899122106	-3.07058842059276	-5.58865006740543
H	1.36790038140051	-3.24916201712432	-4.94567817607274
H	0.44869754173164	-4.55020208536624	-4.12777861595282
H	3.04023083317092	-2.75646485791837	-3.28736206010135
H	2.54205722896054	-4.29618522198902	-2.51206869876445
H	3.68826601316009	-2.59745527612770	-0.98496118747520
H	2.22269876873629	-1.54047613884219	-1.16675318321073
H	-4.63550291380126	-4.27877911691856	-0.81978736700697
H	-5.55153005001541	-2.73986543751388	-0.79561584769215
H	-4.92331585929252	-3.28295380399350	1.45272955437586
H	-3.97295215713004	-1.85263451416445	0.95071589886873
H	-3.19811890875485	-4.12095670480357	3.04087567053758
H	-2.45999001732640	-2.51737854344452	2.72523570792455
H	3.05203793141010	-2.81768171157961	1.44407274388581
H	1.63973697120319	-1.74969337920676	1.07561954098447
H	1.39598669221348	-3.36567473627274	3.07882881344264
H	1.39506442795151	-4.70984316986407	1.89104363709643
H	-0.83365133767706	-4.18785941334042	3.58400267278641
H	-1.11639142520630	-5.22387965752198	2.15146001351885
K	-0.76962490976121	-2.32536400843632	-0.83343020892640
F	0.52161895855276	-0.33798570853304	-0.42680928569965

KBr(18C6)

O	-3.35740821041049	1.66920848234414	-1.95779151054981
C	-4.60057850704106	1.38303117472056	-1.34756483627608
C	-4.54118850674378	0.01756229321106	-0.69968917331491
O	-3.57913661597582	0.03655071604755	0.32569431847455
O	-1.26290538164508	-0.93434861223665	1.70574999712975
C	-0.22751463259838	-0.75332152146872	2.65872511315547
C	1.10693620334839	-0.75423189295501	1.94881943436629
O	1.20481288223817	0.39796342685209	1.13528387247455
C	2.43311498812077	0.56998130102137	0.47527283480640
C	2.46639633429335	1.94843645616280	-0.14649542564527
O	1.46214649417037	2.04341999841963	-1.13892258721445
C	1.39772242824232	3.30239299715663	-1.77314999601684
C	0.35841024187320	3.26629304883376	-2.86961354217877
O	-0.93066838261603	3.12075829716560	-2.31092794636404
C	-1.96235693832709	3.08468429355889	-3.27409839906661

C	-3.30066906834500	2.92850376531335	-2.59027299766973
H	-5.54422762355852	-0.21882191574689	-0.29121622678228
H	-4.29297762576626	-0.75439525831337	-1.45571380393234
H	-0.37160035912470	0.20455801692388	3.19338870576499
H	-0.23860388101988	-1.58271820125432	3.39437260700014
H	1.21623328461061	-1.66896685859748	1.33248492085613
H	1.91449686177169	-0.75280395023535	2.70725984083182
H	3.46529560831090	2.12056895197231	-0.59501579859509
H	2.30300053602021	2.71161895585434	0.63825500793956
H	0.57290914541605	2.42743494706131	-3.56045679920494
H	0.42045924206565	4.20857329711356	-3.45002809852966
H	-1.97024119025051	4.02205246047264	-3.86574313893673
H	-1.80190254624947	2.24226757013697	-3.97536892127536
H	-4.09922812136834	3.01645046445617	-3.35439738893491
H	-3.44796799470241	3.74133655293343	-1.85246661863805
Br	-1.63281130729244	2.83226975694609	2.97556266311487
H	-5.41117806393873	1.38995300614896	-2.10383866571279
H	-4.83688244685360	2.14744010024421	-0.58263298420743
H	1.14623409806229	4.09406644614409	-1.04052622234223
H	2.37667331368481	3.55816296652767	-2.22610627649106
H	3.27689875530792	0.48912522046181	1.18965764912860
H	2.57402519639113	-0.20574580728211	-0.30448208551387
K	-1.19460053696044	1.53546036640475	0.15693902965086
C	-2.56180318335986	-0.90518658875494	2.27313087068497
C	-3.58738582161781	-1.09230589837575	1.17885721150503
H	-2.72623997462959	0.05906257005990	2.79078664711922
H	-2.67635049006500	-1.72795042652524	3.00758519505810
H	-4.58701082738770	-1.20817780938930	1.64240984916594
H	-3.36925635007979	-2.01095010353445	0.59884764616601

KF(18C6)-TBOH

C	-2.89554423480476	-1.98589785883663	-3.63656615302756
O	-1.69293409694498	-2.72543197796172	-3.59016355459960
C	-3.94627096474925	-2.66615578517547	-2.79061974920741
C	-0.65328840434980	-2.14287321573602	-4.36060606715942
C	0.61880470712374	-2.94273720855989	-4.18917359985764
O	1.06384640345656	-2.80867565526274	-2.86172097657102
O	-3.60793242597510	-2.55816844281462	-1.42230343510838
C	2.37542886551428	-3.25556922059659	-2.59653753147828
C	2.75778931789649	-2.83572052026858	-1.19581473733985
C	-4.51511228378796	-3.19338419368228	-0.55279764068649
C	-4.09156637501430	-2.96438503021357	0.88023385681623
O	-2.85830595283889	-3.60612268965309	1.11805681045914
O	1.99214194397962	-3.57261995009793	-0.25806609755836
C	-2.39057009561969	-3.48138677965548	2.44432158459328
C	2.14736736708394	-3.10225069568251	1.07006318700373
C	1.23787337179772	-3.88475807799573	1.98805953863123
C	-1.04840934536165	-4.16595300355257	2.57641506465973
O	-0.10898943255680	-3.50485247734797	1.76705745457783
H	-3.26995438872344	-1.92641063436223	-4.67902916883261
H	-2.72217880485393	-0.95693046260487	-3.27133387149002
H	-4.92589002536986	-2.18234585528276	-2.97905714648295
H	-4.03201302712019	-3.73181827507375	-3.08051007077657

H	-0.48144830743273	-1.10322013143495	-4.02585799681978
H	-0.93535859394513	-2.13626354297379	-5.43315049013788
H	1.37678273432205	-2.54488648210633	-4.89393672996027
H	0.44742717367232	-4.00854192555383	-4.44318667555089
H	3.08589751700894	-2.79464841454099	-3.31196231103864
H	2.44317496359418	-4.35636497818589	-2.70968725015703
H	3.83916026589698	-3.03248837631363	-1.04379360860818
H	2.55539291055871	-1.75652761003289	-1.06598339453850
H	-4.55627753696238	-4.28046113629868	-0.76296180644867
H	-5.53652495316270	-2.78344184979701	-0.69011510901929
H	-4.87593337279533	-3.36775437066857	1.55226066217702
H	-4.00547732706719	-1.87725349761569	1.07617673686969
H	-3.10681697414889	-3.94627515083065	3.15174616785262
H	-2.28935816364713	-2.41234966695219	2.71575481963539
H	3.19438635899911	-3.24987625513904	1.40580205705284
H	1.89607282063571	-2.02519343497254	1.10889361659133
H	1.51732409091193	-3.67077262739303	3.03900691375072
H	1.36058374869748	-4.97226543645029	1.81535160085993
H	-0.74297612817132	-4.12845395500760	3.64179017407389
H	-1.13450088239723	-5.23295714808871	2.28820463536802
K	-0.75059479532652	-2.41301719317985	-0.77263466742426
F	0.85148966607035	-0.57991066395645	-0.14810669643600
C	-0.99840497232828	1.65617676714372	-1.73010754382349
O	-0.65450249443353	0.29255006308890	-1.92721308684274
C	-1.94500667231113	2.05341162554995	-2.86818329834834
C	0.27377720056348	2.51995482802450	-1.77354251650304
C	-1.70131650814979	1.82259783447998	-0.37075321074951
H	0.03980051277613	0.01662121368297	-1.22408841554141
H	-2.24238852496843	3.11169517454138	-2.79219784592368
H	-2.86051022251891	1.43990790157255	-2.84280386689388
H	-1.45624517042869	1.90093192826458	-3.84374819047386
H	0.04421827277103	3.58857432178427	-1.62733643257758
H	0.77811220574483	2.40242577187632	-2.74596911570889
H	0.97308383164445	2.19948296520143	-0.98583473752202
H	-1.98018013568806	2.87254855565603	-0.18039621530178
H	-1.03561379068340	1.48365325455037	0.43820907928221
H	-2.61892086608260	1.21182965249274	-0.34237294772897

KBr(18C6)-TBOH

O	-3.32441329215447	1.90024762929270	-1.78378012356818
C	-4.47049081224019	1.67139602536893	-0.98446420938827
C	-4.50270908847668	0.22275268636919	-0.54915974797906
O	-3.40682593969588	-0.02430794291875	0.30446414909688
O	-1.14705345029149	-1.04753930886925	1.67315843118320
C	-0.14422049158900	-0.88722563578015	2.65658872630841
C	1.20799769061332	-0.78706217349442	1.98747371644244
O	1.27266342602057	0.40789324554256	1.24131074520673
C	2.52034445899219	0.65803310548603	0.62694799054304
C	2.51199073152363	2.03179683228261	-0.00353828287802
O	1.58549310123446	2.06298124034825	-1.07635034157633
C	1.53954255942792	3.32462526057726	-1.72672330912391
C	0.46299703714625	3.31492132716436	-2.78767168836036
O	-0.79710769240241	3.16423941321975	-2.17052679877380

C -1.91117215290362 3.38852041170717 -3.00519790317570
C -3.17704819132856 3.24800648974456 -2.19011811972189
H -5.45487066150913 0.04153652295522 -0.01293973022630
H -4.47249899812962 -0.44952876890761 -1.42967206966229
H -0.33424501206269 0.02641286731667 3.25167647105521
H -0.13924515229888 -1.75413041947354 3.34762565778647
H 1.37274195165458 -1.66446924324719 1.33102791641310
H 1.99350708885417 -0.79513011456576 2.76933190556421
H 3.53339919944433 2.24923274860435 -0.37633966375359
H 2.24366455588137 2.79935833154046 0.74536158936906
H 0.63492360115259 2.49811955128223 -3.51742302859742
H 0.51034500099769 4.27595507202922 -3.33743220978850
H -1.87203773897367 4.41093216640717 -3.43121094501057
H -1.91846372732250 2.66822470457423 -3.84764195768793
H -4.04039803687456 3.55793080038971 -2.81272823350873
H -3.12883159350883 3.90947618371560 -1.30444120803000
Br -2.21193625066216 3.45216551753229 2.02816609334996
H -5.39066182838115 1.89199283783472 -1.56255420263667
H -4.44637029882095 2.32414963724426 -0.09146898495877
H 1.33194698013784 4.11817141584884 -0.98643224672072
H 2.51405964870884 3.53455489978164 -2.21228834095267
H 3.33110851779895 0.62371888813349 1.38164717646994
H 2.73375466146828 -0.11626146986930 -0.13664806698833
K -1.03576424844929 1.39533087388381 -0.01648025388813
C -2.45866087439160 -1.10147102288110 2.20160301518371
C -3.45897359558633 -1.20547084023572 1.07071684195561
H -2.66758046957066 -0.18990865217874 2.79304705921580
H -2.56985895367027 -1.98350073004844 2.86422566973665
H -4.46708915781414 -1.33630944387217 1.51097928955667
H -3.24327493957126 -2.09296704808233 0.44302054009890
H 3.15361354858877 5.75564507244133 1.69633162065339
H 1.79717438913930 4.95874540929181 3.68029484463051
O 0.77030259436176 4.78087926544906 1.20894314075765
C 2.37227797366241 6.50045955656874 1.47649664359284
C 1.01349835710779 5.69552831434375 3.44247240943841
H -0.10924154702214 4.42098836930530 1.46410741875384
C 1.00320523382644 5.99311264714834 1.93551350607062
H 2.36991564080824 6.68683358729929 0.39072385549585
H 0.04431184242092 5.27737990692003 3.75612400115100
H 2.63621231753683 7.43937999522774 1.98788272146234
H 1.20554792117325 6.60886409852107 4.02907689256590
C -0.09330211239966 7.01432369842227 1.59712822665780
H -0.10060910392069 7.22240209349480 0.51534029903333
H -1.08260983082238 6.61974531268165 1.87673754598687
H 0.06715521316231 7.96435880113098 2.13254555616972

KF(18C6)-TBOH-F3

C -2.69991029780347 -2.07572935665225 -3.80791375210995

O -1.55889035270844 -2.90409770290421 -3.69074771218747

C -3.79779571186063 -2.59665836706510 -2.91108318206606

C -0.48572230313788 -2.49368378147258 -4.52146353343818

C	0.71451452870530	-3.38099439206065	-4.27636808840474
O	1.16848094979258	-3.17605333861135	-2.95964603511077
O	-3.43318726736513	-2.42815666501914	-1.55460196503288
C	2.44342705645358	-3.69518130624683	-2.65107870749739
C	2.86185019267935	-3.18617776558821	-1.28980638426871
C	-4.38803462621421	-2.91050840709629	-0.63924099169860
C	-3.93818213949161	-2.60879030042605	0.77177597435026
O	-2.75779246402234	-3.32738913239691	1.05964752116885
O	2.04272929552674	-3.77081834113858	-0.29348489159372
C	-2.28427032053371	-3.14384342004605	2.37807933801485
C	2.25860036132759	-3.21931735061235	0.99490332939981
C	1.30205886930688	-3.85542016573372	1.97627241686868
C	-1.00082267122141	-3.91983681308442	2.57424293290277
O	-0.01289745661509	-3.39643221345674	1.72308889317456
H	-3.07035622831634	-2.07752892741352	-4.85277644211408
H	-2.44803089651690	-1.03614699481431	-3.53465371884060
H	-4.73001789795107	-2.03743078153052	-3.12582027036357
H	-3.98475421268913	-3.66762466306597	-3.12395825927549
H	-0.22018170498350	-1.44436445130930	-4.29854037484110
H	-0.77963039620948	-2.56849839760752	-5.58784369587688
H	1.50390485476795	-3.10812320534775	-5.00538101240686
H	0.45419332517970	-4.44596796765511	-4.44160296665836
H	3.18543846224465	-3.35490335859482	-3.40108516994687
H	2.42876024301005	-4.80348960829721	-2.66591949319604
H	3.92438973669147	-3.45462703796736	-1.11689814205250
H	2.75405760932148	-2.08667912670218	-1.25687738517640
H	-4.52751570995822	-4.00272933670745	-0.76451965909979
H	-5.36930576173637	-2.42276118169456	-0.80852456499806
H	-4.74765051694423	-2.89649460121053	1.47253383272524
H	-3.76378946928156	-1.52067996676652	0.88498196811029
H	-3.03761146031319	-3.49649298828138	3.11100184577636
H	-2.09770701093110	-2.06933577846693	2.57082288588396
H	3.29565116238064	-3.42755287314261	1.32889073816069
H	2.09432359071952	-2.12626879356549	0.95810538141212
H	1.60478519184211	-3.57738298464792	3.00555017042320
H	1.34502829094326	-4.95943693414476	1.89265174588472
H	-0.69550693342726	-3.82319178176011	3.63586771778770
H	-1.16764812649072	-4.99627858713762	2.36848244039797

K -0.61983586655212 -2.53586632454827 -0.90979561660868

F 1.04195415517476 -0.72212220837756 -0.34383194389709

C -0.23838618742383 1.55791975389127 -2.31142773897990

O -0.24102039343218 0.16576212095625 -2.19752801684340

C -1.36960693141222 1.88878681328163 -3.30803671785123

C 1.09736006820651 2.06454246039451 -2.88591298054274

C -0.54211847898443 2.23616927262976 -0.96437375441659

H 0.37664886447716 -0.14228275633036 -1.40514304646488

F -1.47591126077270 3.21759461879354 -3.53573279914370

F -2.57883680807540 1.47480058465411 -2.87368625708647

F -1.17898523068606 1.30692605719798 -4.51185233065485

H 1.10722422200491 3.15707477117865 -3.01846369796985

H 1.30556143462019 1.58508784370785 -3.85308710176341

H 1.89775473279773 1.78658178395971 -2.18498567669262

H -0.54761983102553 3.33344328316219 -1.04749704684006

H 0.23375552552836 1.93320842644907 -0.24660050472300

H -1.51502979861489 1.90173264644302 -0.57629750370819

KBr(18C6)-TBOH-F3

O -3.29241442354217 2.01628208728513 -1.65535543771042
C -4.43800603507025 1.84170225995117 -0.84441468985570
C -4.51511705342093 0.40163545002955 -0.38484842598740
O -3.40356068445702 0.13033504988374 0.43674370401119
O -1.11236931497771 -1.02363081028114 1.67773548618893
C -0.04723360469992 -0.88731145830050 2.60060971650948
C 1.27194530035091 -0.92720361168133 1.86211217212032
O 1.40180903255157 0.23324848485261 1.06880849040123
C 2.61402567070685 0.34110325943311 0.35628984138838
C 2.69818720265517 1.71025546934695 -0.28014394444661
O 1.67857954685801 1.85039794568987 -1.24970722764918
C 1.65700209612040 3.13545919093251 -1.85233412369403
C 0.51996735394780 3.22516353963183 -2.84461398098491
O -0.71408797763924 3.11542484113900 -2.16569985638237
C -1.84964483487498 3.42038342874028 -2.94557028612767
C -3.08806346162432 3.35072677774850 -2.08030168257702
H -5.45770162416427 0.26518706743273 0.18119621451479
H -4.53836551633250 -0.28288618489984 -1.25630004060798
H -0.14155818418789 0.06774962216530 3.15119545023249
H -0.06814873641079 -1.71795353843243 3.33447472752212
H 1.32700638142814 -1.83634081711823 1.23085334653623
H 2.09404179835375 -0.97683234437267 2.60356059749391
H 3.69532866271018 1.82307558984637 -0.75172405105603
H 2.59565035049507 2.49149981834632 0.49646634491420
H 0.60708591075781 2.42951521912227 -3.61156976197044
H 0.58848789265311 4.20396397639497 -3.35724653543272
H -1.76572833139021 4.44374620560168 -3.36093913329274
H -1.93948663326926 2.71197366869003 -3.79320691455357

H	-3.95619005794815	3.70375809196392	-2.67214498280626
H	-2.97148259566619	4.01887630904649	-1.20655412662904
Br	-1.66755523144188	3.09138038881908	2.51873225275881
H	-5.35555439228857	2.08948509819794	-1.41531145587959
H	-4.38454419444737	2.50537449789932	0.03927745299461
H	1.53773727701163	3.91360493842564	-1.07845171526562
H	2.60856695023137	3.31688567214918	-2.39118264025390
H	3.47695537328382	0.21743812687207	1.04048354541233
H	2.67929345502152	-0.44836081296492	-0.41886340338413
K	-0.93540275531493	1.37331309293302	-0.00523202070913
C	-2.39173571159478	-0.97385780186792	2.28536452708320
C	-3.46575316965025	-1.03973933409908	1.22131989460249
H	-2.50128780631940	-0.03390631147835	2.85796867679493
H	-2.51843078890087	-1.83034111782038	2.97800573042763
H	-4.44994872662327	-1.11295601141071	1.72473221201061
H	-3.33636579973105	-1.94269192739028	0.59174381336195
H	2.32877862506214	6.70759038230730	2.23960238255163
H	0.03811283951151	6.45893381224740	3.24188606002788
O	0.74145510817346	4.88754648156256	1.15137370949068
C	1.89244915573867	6.96426431544462	1.26416204118867
C	-0.41681361885485	6.72210890510416	2.27637025748346
H	-0.01735024375792	4.40481809672649	1.56396280740528
C	0.52554548409002	6.28037230161290	1.15098156108692
H	2.57144872474967	6.60704863927391	0.47690626455219
H	-1.38346141259034	6.20295148332361	2.21076888298044
H	1.80654636943340	8.05796918930185	1.18942364122278
H	-0.58540205676826	7.80852585890273	2.24923591145773
C	-0.10431025548514	6.65479615675175	-0.21565897916240
F	0.69060641154191	6.30688368433716	-1.25348803299328
F	-1.28762240901206	6.05016256746433	-0.41966170901883
F	-0.31876533098104	7.98505503918606	-0.32991255829657

KF(18C6)-DIOL

O	-2.46683	-0.43933	-2.88419
C	-3.80838	-0.73575	-2.55184
C	-3.84634	-1.87057	-1.55251
O	-3.22243	-1.45486	-0.36325
O	-1.27686	-1.79698	1.77521
C	-0.57872	-1.20582	2.85987
C	0.89844	-1.13427	2.54399
O	1.10008	-0.18659	1.51788
C	2.43566	0.16214	1.24022
C	2.44011	1.38193	0.34510
O	1.87232	1.04763	-0.90977
C	1.73284	2.15702	-1.77664
C	1.13671	1.70018	-3.08824
O	-0.20678	1.30251	-2.89391
C	-0.86321	0.88500	-4.06950
C	-2.32751	0.64327	-3.77971
H	-4.90650	-2.13655	-1.36645
H	-3.34197	-2.76530	-1.96967
H	-0.96670	-0.18526	3.03072
H	-0.72047	-1.81256	3.77715

H	1.28428	-2.12798	2.23987
H	1.43969	-0.82403	3.46005
H	3.48443	1.72975	0.21048
H	1.85261	2.19082	0.81706
H	1.72857	0.86032	-3.50254
H	1.18254	2.53841	-3.81202
H	-0.78678	1.66599	-4.85267
H	-0.39751	-0.03858	-4.46712
H	-2.84706	0.42488	-4.73456
H	-2.77875	1.55862	-3.34957
F	-2.01901	1.31122	1.58327
H	-4.37437	-1.03074	-3.45830
H	-4.30008	0.15574	-2.11664
H	1.08572	2.92118	-1.30868
H	2.72356	2.61074	-1.98238
H	2.97406	0.41153	2.17645
H	2.97032	-0.67932	0.75499
K	-0.90377	0.17164	-0.36048
C	-2.68423	-1.74960	1.94839
C	-3.36923	-2.33477	0.73490
H	-3.00103	-0.70016	2.09200
H	-2.97680	-2.34439	2.83727
H	-4.44443	-2.47333	0.96457
H	-2.94041	-3.32715	0.49195
H	-1.45533	4.12468	3.00815
C	-0.92279	4.82793	2.35353
H	-1.28401	5.84315	2.57699
H	0.15185	4.78635	2.58967
O	-3.35891	3.56332	1.42707
H	-3.24469	6.17889	1.64722
H	-2.94427	2.66250	1.48901
H	-1.04904	2.47048	1.14670
C	-1.12520	4.45106	0.87616
C	-3.34445	5.77129	0.63135
C	-2.66992	4.39754	0.51168
O	-0.53436	3.16978	0.64853
H	-4.41993	5.65254	0.43023
C	-0.34281	5.42673	-0.01114
H	-2.93972	6.50031	-0.08653
H	0.72616	5.36711	0.24776
H	-0.66732	6.46737	0.13341
C	-2.88119	3.86532	-0.92029
H	-3.96206	3.76042	-1.09940
H	-2.40752	2.88186	-1.04680
H	-0.44394	5.17408	-1.07716
H	-2.46902	4.54117	-1.68614

KBr(18C6)-DIOL

O	-2.51164511591499	-0.52395167150325	-2.80064011686330
C	-3.80252199128026	-0.99301306855660	-2.46410155432083
C	-3.67741504774169	-2.27611397330416	-1.67355536191171
O	-3.02699959002475	-2.00110728333400	-0.45645410247481
O	-1.12797107639530	-2.22848092342349	1.66502668956645

C	-0.54971255819056	-1.70605671439166	2.84798749101870
C	0.90568591673191	-1.38471924637338	2.59632749555545
O	0.99639944636929	-0.31405385423519	1.68062554639540
C	2.30737558400760	0.12330186740108	1.39954754768405
C	2.24491345625382	1.36924029511823	0.54587619750309
O	1.68330801011673	1.05352968479668	-0.71727332870906
C	1.56747790361526	2.17827868329528	-1.57075729152544
C	0.95571051266047	1.75968266597670	-2.88714448987924
O	-0.38979937901526	1.37199723765205	-2.68920164247446
C	-1.08096010703184	1.05320731894282	-3.87671626821881
C	-2.50699385857722	0.67409735243993	-3.54820281422839
H	-4.69456773819831	-2.67589331329333	-1.48939552814618
H	-3.11570117358322	-3.03274464641811	-2.25735293997954
H	-1.08905341645264	-0.79271542506467	3.16165323125656
H	-0.61346390287410	-2.45106529063698	3.66645033671592
H	1.43133381525487	-2.27625241636260	2.20015815464316
H	1.38120915758022	-1.10700162168181	3.55802457976114
H	3.27286885488692	1.76403014803122	0.41733511352981
H	1.63173302777964	2.14093023999365	1.04591790128252
H	1.53221945245053	0.92403203327963	-3.33098518206831
H	1.00597266862468	2.61699209574945	-3.58760844059641
H	-1.09171371012014	1.92408394447951	-4.56229170049339
H	-0.58174050551234	0.21425068814005	-4.40070643863816
H	-3.06765083941767	0.54191624713455	-4.49522682734624
H	-2.99265086111250	1.48963393243403	-2.97775879500379
Br	-3.03223366285969	1.37801861453095	1.78963324098601
H	-4.39294502594989	-1.18811980326869	-3.38158833852204
H	-4.34014096902956	-0.23767614574139	-1.85960428338082
H	0.94042355532501	2.94904296898405	-1.08956026935312
H	2.56886061623858	2.60989292745111	-1.77098837813828
H	2.84245212668629	0.36758115219803	2.33891289029540
H	2.87806129337170	-0.67307087814047	0.88118692075955
K	-1.01392791464510	0.01725492952757	-0.26296507635637
C	-2.50111763919214	-2.54744138620205	1.80534806548799
C	-3.03921808881423	-3.05239697947450	0.48625293404263
H	-3.06843502593226	-1.65196001082626	2.12059421003839
H	-2.63302555694794	-3.33970512587760	2.56977957073024
H	-4.07552527174186	-3.41128215685242	0.64419885792624
H	-2.43388815656529	-3.90607883979913	0.12142187568873
H	-1.48529973104660	4.56268421781289	3.04088996023890
C	-0.73906338244934	5.13782981780545	2.47657053205895
H	-0.90473584426595	6.20425888842777	2.69012905889970
H	0.26462653804537	4.86519771689568	2.83772129465582
O	-3.28083767093025	4.74193517672335	1.28475430569105
H	-2.30815935947923	7.20012118205329	1.30828582507749
H	-3.29258024162585	3.76612762274849	1.34618846155311
H	-1.25638054012428	2.89361277706282	1.24697676657108
C	-0.83565782406519	4.82963299557779	0.97531693425695
C	-2.47468204772548	6.72258292983198	0.33184488933559
C	-2.26563921260233	5.20396532721394	0.40323146523633
O	-0.58171439902155	3.42999784161322	0.77805994139866
H	-3.51815624347971	6.91736765571439	0.04348942656890

C	0.29546728069727	5.54688017261694	0.22890776404800
H	-1.81725222785349	7.19579183607935	-0.41179148371718
H	1.26217673210434	5.15109785346694	0.57670193021555
H	0.28519126166004	6.62884702382004	0.42099413615458
C	-2.48811979837397	4.59532502234389	-0.99259453172874
H	-3.48293765866767	4.89463881738341	-1.35564464423679
H	-2.44785133439161	3.49802146723745	-0.95255310910665
H	0.23537556965591	5.38829444098699	-0.85839287339406
H	-1.73705708089310	4.93822496378892	-1.72096573201600

TS(E2anti) with KF(18C6)
(imaginary Frequency = 715.42i cm^{-1})

H	-1.46350	-1.26270	1.53128
H	0.03975	0.00629	0.11619
C	-1.89946	-0.68133	0.69502
H	-2.22553	0.29670	1.09844
C	-0.83457	-0.47931	-0.35828
H	0.52295	1.09524	-1.91592
H	-0.50874	-1.46131	-0.75571
H	-3.64186	-2.35177	1.85380
C	-0.36396	0.63625	-2.38988
C	-4.02200	-1.67154	1.06565
O	-1.33500	0.32705	-1.40255
O	-3.00155	-1.37199	0.13631
H	-4.37710	-0.74455	1.55598
H	-0.05067	-0.29414	-2.90387
C	-0.92110	1.62849	-3.38103
H	-0.12643	1.86396	-4.11571
C	-5.17929	-2.33531	0.35163
H	-1.18592	2.56635	-2.85913
H	-5.94378	-2.61229	1.10438
H	-4.84324	-3.26530	-0.14849
K	-4.04801	0.17242	-2.13881
F	-5.71964	1.93412	-1.26717
O	-2.05021	1.07180	-4.04410
O	-5.71201	-1.42836	-0.58501
C	-2.58421	1.91052	-5.04556
H	-2.85509	2.89502	-4.61807
C	-6.96026	-1.78431	-1.14464
H	-1.83662	2.08510	-5.84513
H	-7.69592	-1.98015	-0.33969
H	-6.86479	-2.70606	-1.75194
C	-3.80896	1.26325	-5.65408
C	-7.46938	-0.64145	-1.99422
H	-7.44864	0.29405	-1.40637
O	-4.79503	1.10741	-4.65613
O	-6.65569	-0.50091	-3.14625
H	-3.54674	0.28074	-6.09443
H	-4.18200	1.91286	-6.47039
H	-8.51522	-0.85742	-2.29169
C	-7.02065	0.60796	-3.95102
C	-6.07146	0.72066	-5.12269

H	-6.98469	1.53335	-3.34784
H	-6.01083	-0.24655	-5.65977
H	-6.46431	1.47845	-5.82926
H	-8.05021	0.47408	-4.33915
H	-2.53192	2.35051	-0.51305
C	-3.05279	3.28722	-0.30130
H	-4.94385	2.81575	-1.20076
H	-4.16585	3.02604	1.49270
H	-2.41494	3.29806	1.76594
C	-3.27603	3.58923	1.15134
C	-3.92730	3.77391	-1.28626
Br	-0.65382	4.22746	-0.55248
H	-3.47542	4.65886	1.31165
H	-3.59496	3.44935	-2.25017
H	-3.94018	4.84337	-1.25487

TS-S_N2 with KF(18C6)

(imaginary Frequency = 351.29i cm^{-1})

H	0.62016184919050	-0.42599331301461	-0.82390197289843
H	0.31938099110603	0.32902460707462	-3.07966461384439
C	-0.42532340127423	-0.28224066558690	-1.16335069779794
H	-0.77206276070742	0.71275520043509	-0.82679439430195
C	-0.46517597477563	-0.34133039545996	-2.67552219397639
H	-1.09028934756779	0.73776521820926	-4.97169440089972
H	-0.24658614284251	-1.37023874579052	-3.02430565584882
H	-0.38118447511253	-1.36419251603265	1.25990627295359
C	-1.92489971032123	0.17641648893381	-4.50664454127392
C	-1.37511052131651	-1.23367537860938	0.78688249262325
O	-1.73871390949609	0.07727204225772	-3.11258470197958
O	-1.25576428453350	-1.29343809796983	-0.62342214560648
H	-1.78721948719219	-0.25195313730720	1.08758250997248
H	-1.95718813268239	-0.82934986893790	-4.97098701684799
C	-3.21383480017275	0.92450723961847	-4.77567655565677
H	-3.31002146494783	1.09931339397383	-5.86633129228762
C	-2.29410285525404	-2.33591505476292	1.26923441019766
H	-3.17144439729402	1.91099441732352	-4.27564582719829
H	-2.24998540418629	-2.37312625574103	2.37594191725152
H	-1.95449347415005	-3.31666703715591	0.88109962130649
K	-3.92247432109019	-0.69381226308543	-1.53619935460835
F	-3.69360787904758	1.40181578115845	-0.26533704680631
O	-4.31331001505306	0.18105253481464	-4.29589962022084
O	-3.60841904283120	-2.05328768479939	0.84297547669005
C	-5.54614314112102	0.88341409200522	-4.36322619973737
H	-5.50576742488326	1.79696544335332	-3.74240417267357
C	-4.63131956827708	-2.88740262577841	1.33899455354023
H	-5.75614347838930	1.19160615531737	-5.40634855572110
H	-4.48224077948392	-3.08703591443022	2.41842131922279
H	-4.63500057003741	-3.86134725397417	0.81082488561495
C	-6.67975293594884	0.00494838516486	-3.88827581560715
C	-5.95942996426400	-2.18105692953993	1.16399120307217
H	-5.92235116549871	-1.20789765660655	1.68993689711628
O	-6.46913340630884	-0.36703542801079	-2.53985557550561
O	-6.22210668143566	-1.98101340712298	-0.20836233630816

H	-6.76153707217571	-0.90006892935042	-4.52281474708182
H	-7.61514961770836	0.58736966206076	-3.97708177041154
H	-6.76076059530726	-2.79063143672252	1.62862885848842
C	-7.34695386608646	-1.15158869881464	-0.45248842685084
C	-7.58060087678955	-0.99836765499665	-1.93759911558923
H	-7.19206209733648	-0.15519817505060	0.00146317549909
H	-7.75548677844973	-1.98877690473791	-2.40345053675676
H	-8.48729421009712	-0.38026776312970	-2.07530219970705
H	-8.25598401955835	-1.59337496789700	0.00151295113265
H	-6.26817127958057	3.65329701629600	1.01880888189592
H	-4.84953569066096	2.62630175783530	1.39980779611272
H	-5.11691003297660	4.66239010677743	-1.09795234441109
H	-3.62966280573030	3.71339728203525	-0.77881085184381
C	-5.77866776395812	2.69070336838079	0.81158998200694
C	-4.62060725835312	3.69467058668615	-1.25934057456680
H	-6.45264473172649	1.88119070841512	1.12081401923236
C	-5.40000918436901	2.58575765255789	-0.63001818253063
H	-4.48690490774925	3.54385006554037	-2.33843049002592
Br	-7.72657762038536	3.12036439323042	-1.67451913018421
H	-5.62710151380052	1.68996056096048	-1.18700416636304

TS(E2syn) with KF(18C6)

(Imaginary Frequencies = 470.57i cm^{-1}; 16.79i cm^{-1})

C	-3.11576727489862	-2.59679682266103	-3.73414131876947
O	-1.82306223883747	-3.17277018276213	-3.68420704520459
C	-4.05965224007940	-3.38579650374225	-2.85628246829735
C	-0.86443433820491	-2.44390550307576	-4.43056685103927
C	0.49331012402665	-3.08958593507611	-4.28070351561786
O	0.98137593098549	-2.88874867355934	-2.96867043250824
O	-3.72445057836144	-3.19145210454528	-1.49854867056788
C	2.26264196919300	-3.41986671006851	-2.73760058447064
C	2.69923813797728	-3.07257401069411	-1.33222220600668
C	-4.57541814803598	-3.84223788329253	-0.58577552089736
C	-4.12777607314005	-3.53511618838305	0.82503952299212
O	-2.86391804851028	-4.12207506299500	1.05207853717424
O	1.85409137241920	-3.72648347143082	-0.41263858595038
C	-2.36458694123588	-3.90987717007301	2.35726591451409
C	2.15690882991846	-3.48180305366142	0.94360092167104
C	1.24986571705548	-4.32098372966675	1.81450316045991
C	-1.00640337144906	-4.55833676324917	2.48747217017099
O	-0.08074241377189	-3.89211938967810	1.65813481258600
H	-3.50161531078798	-2.61936482219692	-4.77415624519142
H	-3.06732691893063	-1.54791867838526	-3.38977681349712
H	-5.09532144438449	-3.03755708709397	-3.04603284018943
H	-4.01190451643114	-4.46323784528315	-3.11215959837892
H	-0.83021033217747	-1.40035603367534	-4.06860861733669
H	-1.13748057912902	-2.44903488554896	-5.50586745303640
H	1.18698994890276	-2.63345333261509	-5.01543738334809
H	0.42903695360339	-4.17364763238000	-4.50172486753592
H	2.99575329048913	-2.99687430461658	-3.45458765181516
H	2.25897845736653	-4.52058707198323	-2.86800404915404
H	3.75120544673745	-3.39368480575988	-1.18972493580426
H	2.65638106832478	-1.97495917318419	-1.18826479822201

H	-4.56210376733338	-4.93766861652371	-0.75465689878026
H	-5.62046920904940	-3.49260603602390	-0.71330940654009
H	-4.87642311448614	-3.94328281081849	1.53460028486341
H	-4.07432716731927	-2.43996962449060	0.97522981107683
H	-3.04654166311588	-4.36059835394729	3.10719651106146
H	-2.28156203141234	-2.82679698701640	2.56648832587702
H	3.21040740260347	-3.75139970941911	1.16068203734375
H	2.02035719138199	-2.40956503161311	1.18557665838668
H	1.57080571627608	-4.21018066046271	2.87034081846897
H	1.35028629875150	-5.39022213562725	1.53975093745693
H	-0.68691815719265	-4.49500554076346	3.54726673766875
H	-1.06621202074241	-5.63082630916128	2.21407508538225
K	-0.98538844901216	-2.52731547392691	-0.86863378254368
F	-1.46712196882549	-0.40437985851953	-2.19163944822040
H	-4.13190968854039	1.38678866219563	0.29731738903081
H	-3.80453612898031	3.10949741399987	0.59400695552076
C	-3.45389176847864	2.08216791195112	0.81466842753316
H	-3.51847282945471	1.91445274339463	1.89667852200643
H	-1.63069612375000	0.72595373878228	-1.55846993214160
C	-2.05553233827590	1.96248309805750	0.28980667269921
C	-1.81626779439402	1.89562696105357	-1.11540366476188
H	-2.70066848840605	2.15363441705994	-1.71842948108423
H	-1.27576089992297	2.38139668119831	0.92757199006077
Br	-1.40378893956187	-0.16674644672099	1.48004642325625
H	-0.90238053939278	2.40911679867925	-1.44444356035037

TS(S_N2)-TBOH

(imaginary Frequency = 355.94i cm^{-1})

H	0.54215843188472	-1.30231657630348	-0.70019283274409
H	0.52352193495993	-0.35831577788500	-2.92912398374386
C	-0.39215750503984	-0.81679809186684	-1.04697870075747
H	-0.38545039838741	0.22746934528331	-0.67817607397560
C	-0.41136302308058	-0.82434676098879	-2.55878023730952
H	-0.71961267110816	0.55794481445902	-4.80902496861188
H	-0.44162874393600	-1.86734347323051	-2.93117411991159
H	-0.69139396674757	-2.06721987852148	1.26793904524512
C	-1.60961493928534	0.01953569840228	-4.42654268041356
C	-1.58764560535589	-1.55926871964859	0.86096788219733
O	-1.53545531411774	-0.10657318001999	-3.02085090320932
O	-1.52007284031487	-1.50596628881534	-0.55670448840934
H	-1.61976067370285	-0.54141508935827	1.28953866265620
H	-1.62944888575212	-0.98224389134816	-4.89951155536574
C	-2.84867670525922	0.79663682239113	-4.80816918236089
H	-2.81103669317147	0.99993667096149	-5.89724320043216
C	-2.81358439914977	-2.32472284912645	1.29753034154807
H	-2.88204215339382	1.76350271748375	-4.27310890778421
H	-2.81702819140396	-2.34782518514432	2.40198333897688
H	-2.78151911549090	-3.36342051005515	0.91292651580670
K	-4.00115715083167	-0.35662153266845	-1.62035851725301
F	-4.18543162108879	1.86843403505338	-0.38017181384770
O	-4.00149258147834	0.03273373958602	-4.49569431105174
O	-3.97931210902832	-1.67337736212004	0.82388268449401
C	-5.21263804188280	0.66292147304968	-4.87915069838711

H	-5.27941442491999	1.65925405235911	-4.40663913180772
C	-5.17582259992743	-2.15863002390423	1.40110116871167
H	-5.24467030822432	0.78431688844542	-5.98069283394475
H	-5.11983788592525	-2.08242115734403	2.50292741510940
H	-5.32778409201627	-3.22083414249383	1.12469954226154
C	-6.38540260643776	-0.18958075012050	-4.45220221793800
C	-6.34506623873932	-1.32792891337559	0.92597426013903
H	-6.19215924919498	-0.26888984762206	1.20471832629864
O	-6.45513958395768	-0.21663203329302	-3.04198861409618
O	-6.48717328777871	-1.44477942835712	-0.47929443360042
H	-6.28444781838600	-1.21785815092708	-4.85324056493795
H	-7.31192230866257	0.24923988924250	-4.87333299133659
H	-7.26152227755371	-1.68435913458467	1.43640182419118
C	-7.58227495646540	-0.72818788877659	-1.00643755739363
C	-7.59192163048977	-0.86251502190286	-2.51266711874029
H	-7.51142677510626	0.34184053692598	-0.73090140944098
H	-7.59966833322934	-1.93326941409525	-2.79751263988049
H	-8.51806595892744	-0.39887898356234	-2.90735087986931
H	-8.53595794444380	-1.12338597389792	-0.60230438120202
H	-2.19756280665989	2.89461367710977	2.50385858951889
H	-2.57654011896360	3.32464697286328	0.81290091172998
H	-4.78864581077367	3.00012714861168	3.03075586539145
H	-5.20595159226251	3.30267107198562	1.32139642592583
C	-2.47043656833106	2.48718015621879	1.52011191790733
C	-4.98435941789590	2.51825173902027	2.06226802981855
H	-1.65990503378700	1.83309960094711	1.17428328734010
C	-3.77759610079702	1.76845382530874	1.60155366005910
H	-5.86137173541320	1.86759476286898	2.16836061654990
Br	-3.30872431309309	0.43490408071708	3.91280876811428
H	-3.83981613098155	0.71187997642807	1.38636379107481
H	-3.56645643585275	4.89601134031367	-0.81487224081351
H	-3.89728607149536	6.34421167554755	-1.80164112533759
C	-3.64317531190170	5.27282729656192	-1.84703964354230
H	-2.65497579036684	5.16879958447301	-2.32145246275653
H	-6.03656846541006	4.20095256206471	-0.93365163790147
H	-6.40567926984455	5.64941906570073	-1.90974747205204
C	-6.07313311734645	4.59984583363640	-1.95956548575856
C	-4.69668360516241	4.47617773424485	-2.63320705594308
H	-4.26612963056235	2.72024502741057	-1.78680444379637
O	-4.31335132863614	3.10457429387268	-2.70008315971831
H	-5.04758449997215	6.03569189092511	-4.12478176620942
C	-4.76495378600927	4.97234397283604	-4.08020208623823
H	-6.82538298921748	4.02271001513373	-2.52008405867190
H	-3.78705065237610	4.85291867954188	-4.57262064763886
H	-5.50877817433845	4.39380736337250	-4.65083763493049

TS(E2anti)-TBOH

(imaginary Frequency = 592.32i cm^{-1})

H	-2.05892364292190	-1.58639526802954	1.73357864213773
H	-0.31884078075065	-0.33166231959854	0.64116338510511
C	-2.35726987810855	-0.95365227306334	0.87410528892243
H	-2.74708848190689	0.00203605830434	1.27118319419953
C	-1.13965599208070	-0.72577357660333	0.00961307139776

H	0.53842763414420	0.67367671598454	-1.43638840902891
H	-0.80383783448492	-1.69014967494775	-0.41975146006357
H	-4.19534382245910	-2.76358792483021	1.61988683632198
C	-0.38254430388600	0.34710949954340	-1.95747540166601
C	-4.48670390147423	-1.99640386314875	0.87463936552473
O	-1.44007831028606	0.18585290284968	-1.02707901616230
O	-3.35837098254641	-1.60617573530422	0.11058326036078
H	-4.89957971938673	-1.11982481085927	1.40529374242041
H	-0.17055455924689	-0.62395961502425	-2.44704527758324
C	-0.74055869207635	1.38532540242558	-2.99288885723012
H	0.12500940504024	1.50619036393072	-3.67335755515314
C	-5.53843675744302	-2.57827847034832	-0.04225628297112
H	-0.92599536764949	2.37370414696332	-2.53355410842785
H	-6.37524745877266	-2.95676907829719	0.57764278007199
H	-5.12858022671661	-3.43174806254167	-0.61783574012023
K	-4.11049895021554	0.25088985207372	-1.99875196002132
F	-5.84878149308632	2.06870986005848	-0.97410480339777
O	-1.87943279756681	0.95396721283819	-3.72188377522949
O	-5.99236816047683	-1.56682325912051	-0.91647509185305
C	-2.22134414854829	1.83291439488104	-4.78642444410128
H	-2.39407587891565	2.85557451146464	-4.40312701029767
C	-7.14611857946862	-1.87930786840805	-1.66475695573226
H	-1.38445756015591	1.89427979521312	-5.50962460170260
H	-7.97910835306412	-2.15892490215772	-0.98932062467670
H	-6.95339824874935	-2.73532844041698	-2.34118324890574
C	-3.43352024583451	1.30198162547353	-5.51211826070745
C	-7.55511150137201	-0.66335817957996	-2.46516726458968
H	-7.67104767356831	0.20550958324397	-1.79069603297957
O	-4.54688904763036	1.24625446617260	-4.63448660110366
O	-6.56696334406028	-0.39125166734117	-3.44091150395233
H	-3.23388129175380	0.29102130879694	-5.91945994457779
H	-3.65089033302257	1.97632321762576	-6.36276562588853
H	-8.53056226605490	-0.86367891243730	-2.95166755232692
C	-6.85212367255372	0.74108551630545	-4.24063591689406
C	-5.76125685900616	0.91280246525752	-5.27281839955769
H	-6.92765603867627	1.64546463631711	-3.60806427698588
H	-5.64609867266859	-0.02502951043936	-5.85081482427066
H	-6.05708116072604	1.71318432684312	-5.97961496570047
H	-7.81948310313703	0.60768897907925	-4.76455242491893
H	-2.94343818897372	3.47271941549458	-1.49825426041130
C	-3.69376900461065	4.26544094813843	-1.53593140801946
H	-5.42064294027999	3.01307274370656	-1.45933848731285
H	-4.04043718125814	4.83747083443197	0.48115032040360
H	-2.57802458979956	5.56526273964213	-0.23754385529962
H	-4.75638987397590	3.40055197837653	-3.16822356753694
C	-3.61731634419880	5.27969254285190	-0.43999782752168
C	-4.84233240083960	4.04165103936597	-2.28391364511328
Br	-1.66612594293970	5.25102861213058	-3.12362350551037
H	-5.56657216387280	4.86027950141317	-2.37495643975868
H	-4.19565513248673	6.18265590906899	-0.68415386996282
H	-7.24271125922938	0.40826999155411	2.73328446418277
H	-7.63317492724314	1.91661306914080	3.60700965019846

C	-7.12551806635911	1.50318191532236	2.72078963518002
H	-7.63475221836773	1.89214676928403	1.82426885381902
H	-5.01303930754812	0.19490577130899	3.92768307925483
H	-5.33081195293086	1.68649638730172	4.85473167342051
C	-4.91443931204246	1.29186705595158	3.91520113702063
C	-5.63701010858047	1.88196224747557	2.70191998844364
O	-5.00846301653868	1.30172047850936	1.55374768759753
H	-5.41194883056962	1.64926888210034	0.73285400770445
C	-5.47380889188350	3.40823890327775	2.67327654948299
H	-5.92241943041142	3.87516358941934	3.56468473409383
H	-3.84223759272729	1.53981552656808	3.87985016869038
H	-5.96524099557018	3.83671016464968	1.78496888756348
H	-4.40697727343686	3.67813955436670	2.64201468170665

TS(E2syn)-TBOH

(Imaginary Frequencies = 669.23i cm^{-1} of TS; 7.78i cm^{-1})

C	-3.09012884403045	-2.22907999950582	-3.71078807848062
O	-1.86471809721212	-2.92024964652205	-3.62458882125331
C	-4.12983481479739	-2.95817322888823	-2.89191710172472
C	-0.81958454871938	-2.32464127944909	-4.37190573223423
C	0.43775287101347	-3.14780726411868	-4.21466317755926
O	0.90283146189264	-3.04962408062410	-2.88918777714034
O	-3.81193461150708	-2.85523711236999	-1.52211680622524
C	2.12901728436030	-3.70116795626384	-2.64777380618547
C	2.60392734485361	-3.36641229951969	-1.25337398366360
C	-4.68141806978453	-3.57903515659277	-0.67775161236877
C	-4.28618870826236	-3.37687568324075	0.76577020059990
O	-3.02643725718233	-3.97544595472928	0.99769249305513
O	1.70574642429069	-3.93260278944204	-0.31444811742619
C	-2.57872429091213	-3.83639206203077	2.33301634626897
C	2.01429898394349	-3.64025164174388	1.02920148938471
C	1.04331509418818	-4.37128023633480	1.92781434825179
C	-1.24558296342128	-4.52666556434700	2.49804040399216
O	-0.25876849196167	-3.86614574952968	1.73548401059193
H	-3.43542503243123	-2.18232800014521	-4.76408616442294
H	-2.97382824874546	-1.19280055905967	-3.34109759314580
H	-5.12665127020669	-2.51375936471922	-3.09080014026488
H	-4.16673089435595	-4.02180434692329	-3.19987742824368
H	-0.64180782415403	-1.29464768745869	-4.01333898774279
H	-1.09186965302595	-2.29193510754478	-5.44663494254630
H	1.20245475682335	-2.76250646964648	-4.91931364931197
H	0.23627040639365	-4.20565684245212	-4.47886149438760
H	2.89521988995154	-3.36007523422325	-3.37284358056650
H	2.01354960637151	-4.79758533719661	-2.76535135126142
H	3.62019845430207	-3.78661949901081	-1.10910378292749
H	2.64448386865203	-2.26863996827305	-1.13311636838808
H	-4.64947151288060	-4.65690331124098	-0.93285731649626
H	-5.72565294419009	-3.23025794879660	-0.81219256142197
H	-5.05761536680739	-3.84647043460639	1.41016320248573
H	-4.24297089211893	-2.29782032363326	1.00205644015993
H	-3.30390650279140	-4.30534443416291	3.02922865219030
H	-2.48750135704472	-2.76597630437533	2.59396693162728

H	3.04288455437359	-3.97469568742446	1.27557738521657
H	1.96044180498704	-2.54975355749236	1.21229540933396
H	1.35611971133892	-4.23628608123904	2.98312209017234
H	1.07221016570753	-5.45537394651600	1.70034964565168
H	-0.97458983176861	-4.50923050704606	3.57297842124226
H	-1.32192284268965	-5.58712306143871	2.18497457969437
K	-0.99745719005684	-2.47090848147111	-0.72975701769752
F	-0.82835587089752	-0.16189831462938	-1.96747446942466
H	-4.72809493283074	0.32544213639184	-0.91239691401830
H	-5.22189262244749	2.02363435005216	-0.71545921200250
C	-4.60045725624320	1.21655367176184	-0.27896519289416
H	-4.96544473515653	0.99788006146698	0.73138130764120
H	-1.63493811042563	0.74162306486679	-1.62953131759051
C	-3.18224485645363	1.68917538145919	-0.28671629920807
C	-2.43586626178439	1.75646525191354	-1.48341194264738
H	-3.02478895990585	1.61193126818165	-2.40120932881413
H	-2.87321894231079	2.30369599610509	0.55980643604438
Br	-2.25542059909403	-0.13653996200506	1.40078320838732
H	-1.70783328324251	2.57651034181123	-1.54037383641284
H	1.56960063034657	0.92334430154426	1.36961749261399
H	1.83513671400123	2.61385931091715	0.85226424454963
C	1.46520060842745	1.62661895281271	0.52953969593465
H	0.38973700209959	1.71197001721758	0.31227674751351
H	3.81455756007106	0.18114990848414	0.46855730747027
C	2.23489017790141	1.11473156895665	-0.69714336546664
H	4.18332729115455	1.85184932770458	-0.03927065328608
C	3.71224549381649	0.90736291445421	-0.35332284427129
O	1.73856570924133	-0.16839964073954	-1.08213691689069
H	0.81951543041764	-0.08455580399863	-1.42147563621888
H	2.49432754494650	3.08941400060821	-1.61536133572376
C	2.09267860877159	2.09566787901487	-1.87168022763852
H	1.03292473492360	2.21653112921195	-2.14805094839544
H	4.25950486669358	0.51809910554846	-1.22635174501536
H	2.63321343559457	1.71817398223681	-2.75391891106759

TS(S_N2)-TBOH-F3

(Imaginary Frequencies = 354.79i cm^{-1} of TS; 2.58i cm^{-1})

H	0.26323	0.38389	-0.78001
H	-0.04187	1.00842	-3.09648
C	-0.77382	0.42521	-1.16891
H	-1.19682	1.41592	-0.91455
C	-0.74242	0.26254	-2.67190
H	-1.40128	1.14448	-5.03737
H	-0.37113	-0.74677	-2.93854
H	-0.71241	-0.51936	1.29797
C	-2.13769	0.44602	-4.59376
C	-1.70366	-0.51320	0.80336
O	-2.04137	0.46268	-3.18534
O	-1.55524	-0.60615	-0.60194
H	-2.20856	0.43464	1.06871
H	-1.92409	-0.56911	-4.98371
C	-3.52148	0.89255	-5.00520
H	-3.55489	0.97935	-6.10977

C	-2.50500	-1.68941	1.31132
H	-3.73488	1.88353	-4.57061
H	-2.56821	-1.62728	2.41381
H	-1.99687	-2.63510	1.03778
K	-4.28145	-0.34975	-1.66209
F	-4.79231	1.82061	-0.31507
O	-4.48393	-0.04969	-4.56253
O	-3.80311	-1.65666	0.75135
C	-5.81578	0.34805	-4.83231
H	-6.05315	1.28032	-4.28526
C	-4.63424	-2.72317	1.17617
H	-5.94531	0.54268	-5.91569
H	-4.59616	-2.81804	2.27589
H	-4.28828	-3.67116	0.71830
C	-6.76337	-0.75822	-4.43083
C	-6.06683	-2.43848	0.79478
H	-6.40492	-1.51901	1.30517
O	-6.74212	-0.92247	-3.02785
O	-6.18376	-2.30647	-0.61602
H	-6.46943	-1.70235	-4.92991
H	-7.78542	-0.49743	-4.77107
H	-6.69943	-3.27429	1.15250
C	-7.49780	-2.03936	-1.05105
C	-7.53680	-1.99277	-2.56147
H	-7.85650	-1.07709	-0.63576
H	-7.16905	-2.95077	-2.97883
H	-8.58857	-1.86230	-2.88569
H	-8.19159	-2.83164	-0.70525
H	-4.66943	2.70456	3.24493
H	-4.53167	3.45255	1.63471
H	-7.14226	1.93439	2.46848
H	-6.85799	2.56488	0.82223
C	-4.37964	2.52124	2.20063
C	-6.70875	1.69883	1.48604
H	-3.31610	2.25219	2.17646
C	-5.24240	1.45235	1.61649
H	-7.23782	0.83291	1.06829
Br	-5.36925	-0.13643	3.85086
F	-2.38645	3.81191	-2.03337
F	-2.66568	5.81124	-2.81906
C	-3.15094	4.55057	-2.85885
F	-2.92396	4.09204	-4.10969
H	-4.24653	4.58373	-0.33740
H	-4.56297	6.17727	-1.08489
C	-4.84436	5.11467	-1.09075
C	-4.65518	4.49058	-2.47981
H	-4.91602	2.69567	-1.62771
O	-5.05456	3.14448	-2.51047
H	-5.13249	6.29271	-3.63338
C	-5.46150	5.24751	-3.54058
H	-5.90441	5.02956	-0.81079
H	-5.36688	4.75638	-4.51911

H	-6.52124	5.23401	-3.24872
H	-4.82188	0.48603	1.37590

TS(E2anti)-TBOH-F3

(imaginary Frequency = 652.29i cm^{-1})

H	-2.74562902766867	-2.36953077865909	1.87885375280526
H	-0.90306786808580	-0.92354132434902	1.31630025498726
C	-2.92274930973557	-1.59109329694353	1.11012856740679
H	-3.37654963349345	-0.71099287283875	1.60114020072300
C	-1.59451538202064	-1.20662078715636	0.49870133424324
H	0.11650295939627	0.68401168226233	-0.10372584123064
H	-1.15224808811895	-2.06919805585243	-0.03869567531030
H	-5.00200178759169	-3.20896427459365	1.36674664814117
C	-0.58440962167408	0.41796895469640	-0.91906096774002
C	-5.07559251574028	-2.43150029805289	0.58026769216744
O	-1.77616188544484	-0.11884983016723	-0.38528038120311
O	-3.78752474229417	-2.09385621730821	0.10157960362088
H	-5.56422578778156	-1.54321499930164	1.02311678765081
H	-0.08363402752555	-0.32601908387148	-1.57014133930664
C	-0.90539011418993	1.66975614427722	-1.70497526028389
H	0.04332489800012	2.12851607383755	-2.04754247427986
C	-5.91992970220239	-2.97055951555118	-0.55351487202431
H	-1.42013405027159	2.39693087169935	-1.04998679399349
H	-6.88211429093755	-3.32893537718116	-0.13638994198417
H	-5.41406479637385	-3.83391784510739	-1.02937391766772
K	-4.06465452892411	-0.14912903723951	-2.01443688812257
F	-5.51285340909977	1.37221536816452	-0.47782457978728
O	-1.72183932433066	1.34153100961271	-2.81218620961575
O	-6.14657332376045	-1.94746718499929	-1.49885069471534
C	-2.09871000751333	2.45914177996016	-3.59981667731724
H	-2.67483047023303	3.17750502486442	-2.98698019174674
C	-7.00929462914283	-2.29178122004467	-2.56318926451426
H	-1.19925314353543	2.98586299288638	-3.97429344641474
H	-7.96605772274111	-2.68870892760231	-2.16980668547874
H	-6.54878607699201	-3.07707932714845	-3.19452542089893
C	-2.91952014333063	2.00794395303590	-4.78666336106945
C	-7.30334549567298	-1.06018663438173	-3.39195808356196
H	-7.75085548158684	-0.27793941702430	-2.74855978201484
O	-4.09707832693888	1.36361526904144	-4.33890383211547
O	-6.11488450465093	-0.58637915741770	-3.98719766510167
H	-2.32507407900502	1.31587490544560	-5.41563533679054
H	-3.18138597790962	2.89730253257652	-5.38936231053206
H	-8.04841134789686	-1.32477373878127	-4.16850454739802
C	-6.30493787297207	0.55900483219155	-4.81052858482034
C	-4.98744439529826	1.01285693139492	-5.38949814725038
H	-6.75691089270071	1.38526912735818	-4.23356624100428
H	-4.53595270253577	0.21141253878035	-6.00702387346844
H	-5.18821582169313	1.89706959293638	-6.01992180957105
H	-6.99291739139973	0.32015653178058	-5.64428719950263
H	-5.18175910879562	3.01568982763223	-3.01541452043549
C	-5.73198089304986	3.68348531429992	-2.35214677669702
H	-6.09098281139119	2.24740195458715	-0.96112801046399
H	-4.29723396194310	4.54195519117998	-1.03406633072213

H	-4.39448201941207	5.34148094685222	-2.62850592298308
H	-7.44209889999431	2.40562636900981	-2.23982504731919
C	-4.98705478132708	4.87250336600808	-1.83350450574980
C	-6.86278068871692	3.17993662905790	-1.72578442358897
Br	-6.18574517112685	4.56232921824937	-4.95596052948926
H	-7.45365280072236	3.84268398142602	-1.08230950659166
H	-5.66576864814181	5.62550987859607	-1.40662895748243
H	-6.31069928815788	3.19206329075638	2.77356651527873
H	-5.15676212221484	4.55212110513768	2.88114235186194
C	-5.36452959473125	3.59391107874794	2.38371625321356
H	-5.48706525373752	3.77973409643766	1.30677483412984
H	-5.04652803961366	1.93175519867702	4.54300580036915
H	-3.80333329439371	3.20813296853551	4.69179964994903
C	-4.08926365228156	2.29773626548684	4.14593408509714
C	-4.25348591685293	2.56937226329986	2.64854305171231
O	-4.52082942167614	1.33673652066641	2.02282719872795
H	-4.88939157052688	1.45609399928126	1.11786752713666
C	-2.91364340565555	3.11441369694714	2.08711342488737
F	-2.57883998567903	4.30157769901858	2.63507204922896
H	-3.32625363649443	1.52522754573753	4.31554248175458
F	-2.97327444716133	3.30723198686526	0.75110325755024
F	-1.88328874258287	2.27758269227669	2.31070950671683

TS(E2syn)-TBOH-F3

(Imaginary Frequencies = 670.75i cm^{-1} of TS; 11.91i cm^{-1})

C	-3.27717509428170	-2.65432914679164	-3.82383413381751
O	-1.93893823454518	-3.08305487591943	-3.73066411384428
C	-4.14652294037295	-3.54642578026449	-2.96812399563519
C	-1.02759661466319	-2.30033388000797	-4.47999063663370
C	0.35096959266488	-2.90634541099778	-4.36107736126473
O	0.81774392678590	-2.76890951402807	-3.03818684097962
O	-3.83920173899030	-3.34580605319282	-1.60773150256835
C	2.09560118422565	-3.32080329784977	-2.81851641016498
C	2.55180112775921	-2.99536504867800	-1.41653092043292
C	-4.58098085859264	-4.16280691895594	-0.72766218677725
C	-4.23197579093622	-3.83028290130969	0.70371477086795
O	-2.89157381355425	-4.20269100583642	0.96069174349707
O	1.71523714437234	-3.65729166143683	-0.48543801254698
C	-2.47085927427804	-3.92551984242028	2.28197894903992
C	2.06545663402550	-3.41670819935441	0.85931352030597
C	1.16426479322606	-4.22008868443489	1.76726133400288
C	-1.07316870414692	-4.46105908085203	2.48153556764748
O	-0.16250593976451	-3.75111010631485	1.66985890140854
H	-3.63122289379729	-2.71353556861771	-4.87376976791548
H	-3.37172807344086	-1.60332319253494	-3.49116618606840
H	-5.21195260304167	-3.30565426596218	-3.16211345568529
H	-3.98314609254233	-4.60583722817381	-3.24913760464301
H	-1.02101110969624	-1.26193792072583	-4.10127860101633
H	-1.32037640919529	-2.29261422317583	-5.54964513607304
H	1.03256556956349	-2.38689657730029	-5.06483647575292
H	0.31746233120045	-3.97632441160269	-4.64868332275262
H	2.82775525011214	-2.89561965318447	-3.53438514815686
H	2.07180400450782	-4.41871752603482	-2.96981157722617

H	3.60039594465524	-3.33425202295521	-1.29497582803190
H	2.51355443125524	-1.90432575102958	-1.25346558576678
H	-4.37266341136148	-5.23193549462158	-0.93227983743154
H	-5.66772726863235	-3.99587735001754	-0.87339966565797
H	-4.91774250004377	-4.38991569140241	1.37253787934721
H	-4.36869797785601	-2.74885992985721	0.88690036309115
H	-3.14292477666708	-4.42177983179323	3.01145647481056
H	-2.50021487022822	-2.83644247585603	2.47269558272820
H	3.11518333272405	-3.72119669694890	1.04671026248893
H	1.97958351632077	-2.33922430520740	1.09220555207892
H	1.53061575435130	-4.12452396467573	2.80958745834892
H	1.21202807906546	-5.29137582369307	1.48850479691782
H	-0.79801259037499	-4.35540877216945	3.55045468893421
H	-1.04514138247519	-5.53954282854029	2.22910737332410
K	-1.09185199809973	-2.43356798794313	-0.79320997920146
F	-1.34130956818570	-0.04757641995143	-2.13537071652589
H	-0.75449483915117	2.01797144805873	0.05074073119212
H	0.63317956927992	3.12234626421175	0.28001758502004
C	0.28531224428551	2.21967140693692	-0.24225901559755
H	0.31882350662995	2.40146437049749	-1.32613188054708
H	0.00547516002791	0.52263059805721	1.90142566698619
C	1.13480440966929	1.00258383755892	0.13823788628205
H	1.41526223395420	1.58068466581243	2.23321495398938
C	1.05518558243668	0.72467286468347	1.64388366615393
O	0.74105613565767	-0.15249653116170	-0.57147848046030
H	0.02006466732116	0.04976372462936	-1.21923912106482
F	3.12913776773345	2.32830853703855	0.38774559149691
C	2.60795491309245	1.26288616319396	-0.26096455854341
F	2.74650990357079	1.50170144731468	-1.57745863007821
H	1.65481215132809	-0.15882930138251	1.90589568976286
F	3.40102527049102	0.20883160118005	0.02918455031558
H	-4.26315202091548	2.46018225297160	1.22574801384580
H	-2.98542662388506	3.08268285885518	0.13355772435435
C	-3.99074488509302	2.63786102275115	0.17901118761298
H	-4.69355257048196	3.37967379507350	-0.25082462710612
Br	-2.95817717682352	-0.22155749361718	1.13207096418272
C	-4.08794603724980	1.40626450780362	-0.65966904216805
H	-3.06680717221890	2.26563762940258	-2.31307366585258
H	-4.90953774109454	0.72766346983507	-0.42583872356375
C	-3.48558665964344	1.32252388971453	-1.93363959306698
H	-4.05485321980997	0.74971306963988	-2.67855492966213
H	-2.40197465616226	0.61002122356172	-1.92834215975216

TS(S_N2)-DIOL

(imaginary Frequency = 337.32i cm^{-1})

O	-2.31738	-0.57599	-3.03174
C	-3.65603	-0.91853	-2.71580
C	-3.68724	-2.20025	-1.91525
O	-3.00948	-2.01492	-0.68836
O	-1.25073	-2.28446	1.50777
C	-0.75105	-1.89850	2.76870
C	0.68439	-1.44545	2.63210
O	0.74673	-0.29185	1.81843

C	2.05195	0.22756	1.65730
C	2.02382	1.42562	0.73713
O	1.66841	1.01287	-0.57253
C	1.71153	2.06903	-1.51824
C	1.23802	1.57296	-2.86535
O	-0.13015	1.22829	-2.78801
C	-0.72846	0.90273	-4.02514
C	-2.19340	0.59792	-3.80711
H	-4.74317	-2.46965	-1.72640
H	-3.21198	-3.01786	-2.49276
H	-1.36534	-1.08162	3.19514
H	-0.78682	-2.74842	3.47919
H	1.29554	-2.25693	2.19051
H	1.08762	-1.22802	3.64105
H	3.03408	1.88127	0.73058
H	1.30610	2.18358	1.10170
H	1.83691	0.69914	-3.19080
H	1.38833	2.38178	-3.60771
H	-0.64052	1.75310	-4.73013
H	-0.22327	0.02847	-4.48118
H	-2.68206	0.46459	-4.79299
H	-2.68016	1.45194	-3.29825
F	-2.66578	1.66129	0.14694
H	-4.24417	-1.06652	-3.64277
H	-4.12989	-0.10146	-2.14087
H	1.07084	2.89976	-1.17450
H	2.75048	2.44117	-1.62355
H	2.46246	0.54141	2.63761
H	2.72215	-0.55088	1.24249
K	-0.95213	-0.17837	-0.47092
C	-2.59119	-2.76085	1.55131
C	-3.05760	-3.14450	0.16794
H	-3.26962	-1.99276	1.96342
H	-2.65673	-3.65294	2.20421
H	-4.09772	-3.50425	0.25911
H	-2.42787	-3.95559	-0.24868
H	-2.94539	4.54387	1.66411
C	-2.03191	5.15332	1.61640
H	-2.32616	6.20997	1.69998
H	-1.39610	4.90671	2.47993
O	-3.29780	4.15653	-0.82797
H	-3.38038	6.69171	-0.20318
H	-3.02096	3.23679	-0.63969
H	-1.46072	2.88425	0.44034
C	-1.24998	4.87426	0.32313
C	-2.77340	6.43206	-1.08144
C	-2.17495	5.02685	-0.95261
O	-0.73193	3.53880	0.37071
H	-3.43639	6.45676	-1.95934
C	-0.01406	5.77843	0.27010
H	-1.99742	7.19937	-1.21822
H	0.58980	5.61571	1.17584

H	-0.28727	6.84254	0.23008
C	-1.41704	4.67255	-2.24355
H	-2.12144	4.71794	-3.08793
H	-1.00636	3.65442	-2.18767
H	0.61798	5.54638	-0.59895
H	-0.59446	5.37399	-2.45084
H	-3.25740	0.51193	2.72946
C	-4.04001	1.17272	2.33550
H	-4.94407	1.05130	2.94950
H	-3.70567	2.21858	2.41868
Br	-6.10071	-1.27574	1.37805
C	-4.36203	0.87412	0.91030
H	-3.99353	-0.03063	0.44539
C	-5.34164	1.73635	0.19214
H	-6.31896	1.66431	0.69098
H	-5.01683	2.78757	0.23278
H	-5.46790	1.43595	-0.85572

TS(E2anti)-DIOL

(imaginary Frequency = 583.14i cm^{-1})

O	-2.37829166737938	-0.61560749612665	-2.97249510230247
C	-3.70158729250454	-1.05439641112421	-2.71823523550473
C	-3.67124443602479	-2.34003238593407	-1.92332333628248
O	-3.09166392256129	-2.10404059548078	-0.65702083870808
O	-1.31697263409799	-2.29446141781101	1.52788376105308
C	-0.82423758350238	-1.93070152394246	2.79816881905740
C	0.60351820899264	-1.45343942701538	2.66975881403433
O	0.65312249753081	-0.29041057666905	1.86509399904594
C	1.95927434002477	0.22339946657800	1.69196739191359
C	1.92896850121275	1.42612044345395	0.77872823532118
O	1.54016740651025	1.02476801677656	-0.52466859098781
C	1.60315185566907	2.07833290236897	-1.47240539331284
C	1.11730409371435	1.58808593128582	-2.81637002616364
O	-0.25401018396795	1.25488493968099	-2.73215764395880
C	-0.84989432051230	0.92095811237783	-3.96841193944389
C	-2.30178215516752	0.56497984552599	-3.74506096283004
H	-4.70728924672938	-2.71141749229860	-1.80692122857747
H	-3.09111771302000	-3.10718791926561	-2.47360418537537
H	-1.45354364313498	-1.13756088592697	3.24762935825250
H	-0.84296801082812	-2.79804858313047	3.48723589618541
H	1.22687063721090	-2.25052380629924	2.21967022165915
H	1.00355362955713	-1.23894028469176	3.68029027706540
H	2.94523777347589	1.86716496841270	0.75336904300992
H	1.23106006149850	2.19346838282373	1.16165597316042
H	1.70514445757830	0.70825658721458	-3.14524593460275
H	1.27136344281166	2.39528957636612	-3.55955470389195
H	-0.79261546886687	1.77889478934564	-4.66731220722807
H	-0.32003690565389	0.06651355470999	-4.43348879808355
H	-2.78825922082399	0.41588805044731	-4.72939066933519
H	-2.81895061929769	1.39773486613105	-3.23081311253381
F	-2.91997502298267	1.81076595505618	-0.08565147476633
H	-4.23445145837657	-1.23773494674070	-3.67178305436623
H	-4.25789213550024	-0.27581922662278	-2.16263569857567

H	0.98055223085594	2.92266618186023	-1.12934796669984
H	2.64931789388274	2.42819643006394	-1.58108280481220
H	2.38396872352195	0.52839133475638	2.66888470442672
H	2.61947634594858	-0.55654576862262	1.26439076030317
K	-1.08135271902809	-0.17130248332230	-0.40719665213484
C	-2.62109062419213	-2.87034272493375	1.56763358221923
C	-3.06067088638815	-3.25634373688883	0.17807126402772
H	-3.35528114575601	-2.17279903841757	2.00776320373152
H	-2.61637082125293	-3.78014429622005	2.19747339256263
H	-4.07344526523730	-3.68845273296898	0.26355998192935
H	-2.37308491188849	-4.00755082562276	-0.25827405329478
H	-2.88856194321946	4.77711587900027	1.70910314708046
C	-1.93606011916299	5.32305104435713	1.65752063005253
H	-2.15512842164841	6.39555370564219	1.76409750329872
H	-1.30510018870169	5.01763681026756	2.50563846747299
O	-3.33790861866389	4.55863445624087	-0.76876127754017
H	-3.09345319151370	7.07669421621971	-0.13105778450606
H	-3.17915585294077	3.60051015082073	-0.72366033105182
H	-1.59285985831447	3.06028407138650	0.38141504624412
C	-1.19695834684733	5.01509326554338	0.34716746377364
C	-2.53956059470375	6.75179373868953	-1.02257627234377
C	-2.11587950152460	5.28351468667412	-0.91293596443119
O	-0.80580116264323	3.63211375579742	0.35312127436180
H	-3.20881664094611	6.86637326111190	-1.88806655755796
C	0.11939144708651	5.79677209271396	0.30299077801719
H	-1.67567723744302	7.41645078075397	-1.16688696204857
H	0.72894659133655	5.52490373726454	1.17829364311906
H	-0.05076205635915	6.88214932658305	0.33379901273895
C	-1.43049955656027	4.84503361026759	-2.21786582545005
H	-2.13675316528881	4.98227666949448	-3.05042285278861
H	-1.14253249604037	3.78433292387934	-2.17486976844951
H	0.70416848797827	5.56163642477153	-0.59803370971693
H	-0.53038926153190	5.43973148867098	-2.43633312389043
H	-3.50460500134834	0.20817096921102	2.16689055083267
C	-4.34948417655323	0.80486168557452	1.80671650069037
H	-4.69695445029374	1.58498453978208	2.49365799628898
H	-3.64435125428318	1.42620773757505	0.72058905512031
C	-5.24613575706269	0.22140728832224	0.93160713675658
Br	-6.28625653971546	-2.26641217003268	1.90640758285693
H	-4.88677136005901	-0.59956729480113	0.31060878181301
C	-6.52318408361481	0.87422406901623	0.51747528265159
H	-6.89599171088197	1.57046164494007	1.28261784018643
H	-6.35035734757548	1.44730002795914	-0.41360241162439
H	-7.28909871627999	0.11529965714325	0.31317808285731

TS(E2syn)-DIOL

(Imaginary Frequencies = 654.51i cm^{-1} of TS; 2.31i cm^{-1})

O	-1.92901164036788	-0.51340738562822	-2.96578523418315
C	-3.28389674035867	-0.90774742115230	-2.92864033373180
C	-3.38595910323727	-2.30099143082360	-2.35406957975221
O	-2.99634017182717	-2.29127555510301	-0.99896488849594
O	-1.14852511237944	-3.09516098261582	1.04615708375933
C	-0.59367183465618	-3.05599845235308	2.34898005303070

C	0.83901442578588	-2.58187921817174	2.27798417186671
O	0.87242021243042	-1.23908787532792	1.84912010314265
C	2.13971581270198	-0.63052999110503	1.92464218699517
C	2.03351722456932	0.80256360297452	1.45379921764726
O	1.68604696090881	0.80482686055495	0.08700415578310
C	1.63370403039848	2.08493011938738	-0.51570582698096
C	1.37178059256284	1.90661020989458	-1.99346062332727
O	0.09184334542925	1.34277405397027	-2.17251374044646
C	-0.26571925332859	1.11548339710489	-3.51644676353735
C	-1.72663222464726	0.73752781747108	-3.59146637905118
H	-4.43251832776199	-2.65445276102556	-2.45279235207246
H	-2.73967235801937	-2.99021095745612	-2.93267058652482
H	-1.17948066915309	-2.37234431947457	2.99025489829934
H	-0.61027073535179	-4.06950370666658	2.79910050132171
H	1.42557054867063	-3.22078933428230	1.58730097214704
H	1.28646250319243	-2.67375920978852	3.28798599697805
H	3.01066890066089	1.30379514859419	1.60741530433022
H	1.27496998210613	1.34212778497504	2.05334485430017
H	2.14689624448120	1.25137959288723	-2.43858383227885
H	1.43018661457060	2.89506701801992	-2.49161548370105
H	-0.11109328808963	2.03313950986434	-4.11819031696046
H	0.36175741590544	0.31354866103158	-3.95425290661266
H	-2.02823140382886	0.68490675625299	-4.65744236867918
H	-2.33795302063585	1.51464246070091	-3.09831284926342
F	-2.99069341835617	1.62004211847876	-0.38915872670408
H	-3.71189869649357	-0.91733834798831	-3.95151428287282
H	-3.87182096593232	-0.19655709844056	-2.31810626528900
H	0.83566531152617	2.70522219514281	-0.06570975118398
H	2.60096293512293	2.61092478050965	-0.38608072621175
H	2.51331823135317	-0.64043297228484	2.96848162608925
H	2.87108496552135	-1.17656592076137	1.29561048091082
K	-1.14952087238951	-0.25478137121495	-0.01680922568766
C	-2.51042683761542	-3.47210618721736	1.02953106541124
C	-2.98936593465901	-3.57003062571402	-0.39998084383296
H	-3.11627839924402	-2.73643712209595	1.59169002750612
H	-2.64015915492318	-4.46436935954647	1.50790508648589
H	-4.01044133670228	-4.00229323170138	-0.40666862848345
H	-2.32900826731470	-4.25224337320123	-0.97084471370845
H	-3.90999873047126	4.48509521613013	0.85146603337766
C	-3.11739602640373	5.24278508277854	0.92719047333085
H	-3.59259377900239	6.23420337764567	0.88668079003949
H	-2.61958089986759	5.13852269784716	1.90281981354445
O	-3.74969092577030	3.96842011903813	-1.62771293007926
H	-4.32317665961409	6.48010459977857	-1.21523520086742
H	-3.38251400013950	3.11533174851462	-1.32648006789573
H	-1.96186332268056	3.07634871261114	0.05500358870716
C	-2.07596992118090	5.05858199130240	-0.18797185968292
C	-3.53374968769705	6.28088331599273	-1.95322567300410
C	-2.76305689592206	5.00075813899570	-1.61311460789369
O	-1.35676951787015	3.84237564206126	0.03576310828671
H	-4.02139362794747	6.15213968034488	-2.93145924635462
C	-1.01889996413594	6.16317243440174	-0.08649932549325

H	-2.87194971576265	7.15706334165264	-2.01338443014697
H	-0.56357526977767	6.13047765769476	0.91480075487286
H	-1.45441147317611	7.16213579001218	-0.23197250379023
C	-1.72642514416190	4.71670091069556	-2.71277018300797
H	-2.25379030859974	4.55687834255599	-3.66555992382513
H	-1.14581034087001	3.81580518025714	-2.46880654175178
H	-0.21485224098587	6.01821506239402	-0.82199295568509
H	-1.02482449080339	5.55377504916169	-2.84832390902093
H	-4.78679475551100	1.92560828085090	4.12009136035225
C	-5.18746884905432	2.22403256686171	3.14457100016441
H	-6.24619467378884	2.52711768026196	3.27141262374701
H	-4.63907455802336	3.10312181741582	2.77505850940006
C	-5.16326725946452	1.10927429446132	2.15644221699798
C	-5.24437745062076	1.33207192740323	0.77366092739714
H	-5.56295299679858	2.33950040192182	0.46878391741811
H	-4.04409845751158	1.42824100343190	0.27995717781844
H	-5.65710260759223	0.51249025369208	0.17214849115759
Br	-2.69254185444154	0.10909713259575	2.94789746067278
H	-5.33801008497714	0.10577267256221	2.54588055478287

TS(S_N2)-BDMb-F6

(imaginary Frequency = 348.26i cm^{-1})

C	-1.70754310426463	-1.24604927119958	0.26323773460939
C	-1.20939913374843	-0.56492535813305	-0.98922955839885
O	-3.08688327851088	-0.99013491088124	0.44865228255381
O	-1.82930395008437	-1.13842339665553	-2.12438131326027
C	-1.41366363035079	-0.55255734292674	-3.34830121348494
C	-3.61280988032080	-1.60208588575216	1.61223587922253
C	-2.05569738767078	-1.28083559337486	-4.50555391670868
O	-3.45657759037662	-1.10918595752238	-4.45009850893466
C	-4.15503872005898	-1.58582066475989	-5.57763338538903
C	-5.11222679041117	-1.43270721928200	1.66800041843578
C	-5.63340194127116	-1.32806256087051	-5.39403852380646
O	-6.10639844093144	-2.09968180161642	-4.30654756211035
O	-5.69379707413109	-2.10266805040045	0.56176675167670
C	-7.50155744558888	-2.01499114528050	-4.10049186759459
C	-7.10153806086542	-2.24190149561962	0.64903520447459
C	-7.87977323266978	-2.85908197203662	-2.90463882553265
O	-7.33617340003742	-2.28701414305182	-1.73446356644360
C	-7.59991735399586	-3.01532307927433	-0.55017192113612
H	-1.12722667308748	-0.86492473041038	1.12663002403815
H	-1.53353543466284	-2.33766323265423	0.19347379888004
H	-0.11041982034815	-0.69730258320053	-1.04566281086725
H	-1.41232891728372	0.51908851194071	-0.94097990809300
H	-0.31305556346050	-0.62483409313879	-3.45112611223773
H	-1.70296196930259	0.51157236454742	-3.37482606357738
H	-3.16457744688740	-1.15061951062282	2.51916809291146
H	-3.36323297998868	-2.68103894612811	1.61022614275983
H	-1.65413999592606	-0.85879129628055	-5.44787411312202
H	-1.80141758486323	-2.35895256278854	-4.47799120815293
H	-3.97645465905857	-2.67065519653583	-5.71517025348272
H	-3.81115464992315	-1.06483053725183	-6.49344545849821
H	-5.47378928567331	-1.87287304174510	2.61733777180698

H	-5.40351167053894	-0.36593490871462	1.67344231724605
H	-6.16299390340645	-1.60945807333560	-6.32596089863313
H	-5.81080845935207	-0.25193448117031	-5.20520575814861
H	-8.04546712575118	-2.39021042236164	-4.99045258500784
H	-7.80199413154078	-0.96397803148249	-3.93179786670229
H	-7.58696736423939	-1.25066220717316	0.71448963824998
H	-7.37289916872668	-2.80409219695119	1.56441432990800
H	-8.98460353442510	-2.90778816125789	-2.83244102706607
H	-7.50289739644343	-3.89165028229289	-3.04085444716421
H	-8.68905082151441	-3.18150369515789	-0.43499363403620
H	-7.10746353869817	-4.00640417426167	-0.59944709560671
K	-4.75227750101712	-0.92122834136474	-1.96773896796777
F	-6.06143095721999	1.31490714345616	-2.56482730161397
H	-6.46879974751268	3.73440729451810	-0.59744229831427
C	-7.31088881817992	3.26234479213806	-1.11353389275308
H	-8.25107490400362	3.67276925053828	-0.71654248974970
H	-7.26850054555904	3.50199084004940	-2.18899719721098
Br	-7.93830220060072	1.63112270830613	1.67139502270517
C	-7.30668883228252	1.78017851126007	-0.97833325586882
H	-6.49741876377460	1.28906058443747	-0.46070053621969
C	-8.46781081897123	1.00987894497291	-1.50620767189892
H	-9.38808684943404	1.32304985004464	-0.99216349565814
H	-8.57644838722123	1.25297247785593	-2.57563389195108
H	-8.33281730722707	-0.07141629004013	-1.39511082655332
F	-1.44682255896375	3.27169473978330	-3.94892006038598
H	-3.84606024948282	4.07468080533726	-4.44101127385888
C	-1.79793521265403	3.56511122256500	-2.68331633434617
C	-4.06225839141096	4.36224635879309	-3.40297472382007
F	-1.38734790104822	4.82477099027492	-2.44364552822897
F	-1.07508882725036	2.75508233372321	-1.88402205805921
O	-3.60109316000440	2.07150609549979	-2.77818569894236
H	-3.77187195384021	5.41191587836439	-3.26158300137444
C	-3.32304649037453	3.40977984113519	-2.44821370096086
H	-5.14353991678814	4.27106142212623	-3.23182248372307
H	-4.58647873476016	1.89386884830728	-2.73529585398948
C	-3.63049776435587	3.69299579914339	-0.96572998860226
H	-3.46199271385460	1.61474255130081	-0.40317940056417
H	-3.84304268731619	5.84412359578764	-1.13434378894752
C	-3.68570132316871	2.62395868517817	-0.06092774406019
C	-3.89681701010566	4.97703329588942	-0.47464474863933
C	-4.05678292033261	2.82349635889866	1.26566392212230
C	-4.26386497582296	5.18035632281593	0.85969787022895
H	-4.12906560031642	1.96884341425962	1.93926147465667
C	-4.38161176088291	4.10022982619503	1.74552364140356
H	-4.48017979442663	6.19663997790802	1.18677262101921
C	-4.81796283356424	4.20774072993787	3.21815470946475
H	-6.24722017181543	2.82461552961187	3.02103066836022
F	-6.78567269621517	5.45654012928948	2.61979683587375
H	-2.97516972558812	3.42772786273475	4.00614794449628
O	-5.51233200864966	3.05670323359730	3.62952674913840
H	-2.98635192325979	5.20945218190070	3.87868963398743
F	-5.08817011850212	6.60905832019165	3.32715888417145

C -3.59064331759003 4.32845819958941 4.13366573761067
C -5.74461592104485 5.43064319738225 3.46769674187615
F -6.24014052394907 5.41860859756613 4.71174139036865
H -3.90821266926256 4.39127722580470 5.18427338320322

TS(E2anti)- BDMb-F6

(imaginary Frequency = 782.54i cm^{-1})

C -1.77739339254121 -2.07902599047081 -0.15689105994630
C -1.34721111622507 -1.05613603374402 -1.18075588045712
O -3.11286680582248 -1.85323319442104 0.24151524405462
O -2.12523787506112 -1.19197253464958 -2.35362673792871
C -1.68550380017673 -0.37084517914087 -3.41766561056584
C -3.56496768756076 -2.79640086782540 1.19805835548970
C -2.59059324180625 -0.55108725151560 -4.61396580629429
O -3.89985435926384 -0.13804582275253 -4.28824219570246
C -4.78006918723922 -0.08441043157132 -5.39368411171334
C -5.05524474832516 -2.67224530186044 1.40541515278206
C -6.19304450729042 0.14173197480003 -4.90707683492765
O -6.60573007322032 -1.00004505595619 -4.17639087034332
O -5.70975857245032 -2.94403420427825 0.17625686547318
C -7.96273867883918 -0.98393441483285 -3.77812115754407
C -7.07519710737984 -3.30138488089857 0.30875262291307
C -8.26630544488146 -2.25663665603965 -3.02011613354307
O -7.57210907025517 -2.25644983895498 -1.78917847722474
C -7.67308192451034 -3.47103175631821 -1.06843415617950
H -1.09610753507789 -2.01011500668160 0.71476627988950
H -1.67828876210161 -3.09685710756932 -0.58228963104413
H -0.27595796124242 -1.22479737708690 -1.40832417367988
H -1.44748625058649 -0.03488654688537 -0.76901668332118
H -0.65199116268061 -0.64392452902104 -3.71101829832866
H -1.68277923068732 0.69015262101616 -3.11032580626051
H -3.04744620831231 -2.64034118946658 2.16502231744767
H -3.33020355173214 -3.81975909483631 0.84653180973467
H -2.18686995955352 0.05609463748734 -5.44819241119616
H -2.59466214816796 -1.61098173735225 -4.93701224002600
H -4.73334792002337 -1.03290430005844 -5.96340602494658
H -4.49009040962400 0.73998544081352 -6.07438890539404
H -5.35752571771817 -3.40442781101218 2.17728566703254
H -5.33878287683830 -1.67303389113321 1.78441186724736
H -6.84973207466151 0.29464871221251 -5.78612146908635
H -6.24917749344906 1.04339314904962 -4.26948204505687
H -8.62300900999929 -0.93162196872808 -4.66658841174444
H -8.16256201855200 -0.10003453840736 -3.14507003815774
H -7.62225249861996 -2.54103697121707 0.89493078739768
H -7.16823410397096 -4.26174053241400 0.85334206949178
H -9.35842180344362 -2.32423179446016 -2.84480365359315
H -7.96607373133473 -3.13115590901714 -3.62983156347152
H -8.73404900463445 -3.76839043567191 -0.95770735804345
H -7.15031531121666 -4.27697610872628 -1.62048748832709
K -5.05361517786950 -0.82251534417014 -1.74686530197417
F -6.59783874158553 1.46228372929611 -1.84077748672480
H -10.54324945560593 0.60319577159275 0.37222591530246
H -8.74372907530407 2.45038340423952 0.31928720803743

Br	-8.28653864250015	-0.60809236846613	2.98337090443452
C	-7.98611908696767	1.65858836190724	0.30536888129260
C	-9.78406047963160	-0.10366933245271	0.00809384062301
C	-8.40523352734050	0.33770625369990	0.37383748678802
H	-7.05113059724234	1.91277474104674	0.81124005197720
H	-10.00425629820258	-1.10227889010157	0.40444087642264
H	-9.85920939290265	-0.14523368720713	-1.09497477194914
H	-7.36536395702186	1.55122755847278	-0.91025165659365
H	-7.63696273865681	-0.43332002367423	0.37524430494625
F	-2.59477946264495	2.95202265324975	-2.42754097325948
H	-3.09761427905062	5.34742731512641	-1.90688244904650
O	-5.14426752236280	3.75035087305823	-1.79850513308587
C	-3.48793109810697	5.17812977492884	-0.89388363155648
H	-4.24502449445198	5.94410397715050	-0.68478405046298
H	-5.66191507921958	2.91212423389567	-1.76492585659998
C	-3.07493154112837	2.74385418425359	-1.19159699083598
C	-4.14787338248187	3.79328173631470	-0.80630380369160
H	-2.67378371132293	5.27930423413948	-0.16290375329516
F	-3.56934998338928	1.47767226093617	-1.18857477159434
F	-2.01063095562280	2.73993256916411	-0.36201238199850
H	-6.12735522021688	5.18349346861817	0.34048330395877
C	-4.73327027705064	3.55233484103984	0.59901323075835
C	-5.76883212930904	4.40329077499211	1.01496443058904
C	-4.33114496519520	2.54354889029177	1.48728136391939
H	-3.53130369096676	1.85052171792170	1.23117940769509
C	-6.37911353034178	4.26063797140369	2.26182663306420
H	-7.17724439517979	4.95187633801503	2.52932059100717
C	-4.95572496422451	2.38764845565385	2.72683376343884
C	-5.98972842081709	3.23772042832798	3.13978125586050
H	-4.64516350350966	1.57583651860918	3.38650350125059
F	-8.03560023964749	4.92867416500790	4.62923075334029
F	-8.84989208941201	3.11056530716900	3.76768490704061
C	-6.57332158440679	3.00181696248039	4.54777697114792
C	-8.01046109376700	3.57313659336940	4.70699153486415
H	-5.56682005248458	4.74477259287228	5.41607901351297
C	-5.67050482093510	3.66676148932228	5.59961521284795
H	-4.67780793012398	3.19970980552061	5.54626979190216
H	-7.15889610330529	1.10873942032482	4.24049687732669
O	-6.61153346316217	1.63655107386595	4.86646523006419
F	-8.52612957473013	3.25391470016951	5.90111951130466
H	-6.08144893352214	3.50153422822012	6.60546245304537

Dimer: [KF(18C6)-BDMb-F6]$_2$

(Imaginary Frequencies = 14.37i cm^{-1}; 11.50i cm^{-1}; 7.94i cm^{-1}; and 6.75i cm^{-1}.)

O	-3.53918060508493	3.99734128082364	-2.28884811865769
C	-4.29341869901903	5.15162456631719	-1.97578888217683
C	-5.35407493676705	4.80791903218366	-0.95313817386877
O	-4.73138027505358	4.44038924324596	0.25441312709777
O	-4.23021522124681	2.70049331753671	2.47136475491127
C	-3.43343028783374	2.30238430780812	3.57297474882636
C	-2.90812117794185	0.90519787526147	3.32994068618921
O	-2.04536008725444	0.93052346011071	2.21673827630931

C	-1.38494493969680	-0.28141916725069	1.92835434973651
O	-0.76670384068465	0.47015317669799	-0.26731405067311
C	0.24056234062757	0.87520636608654	-1.17098875920736
C	-0.38492019939673	1.28479599102520	-2.48562688089139
O	-1.13802363378719	2.46860944781765	-2.31610916871704
C	-1.76515295680245	2.93501539564481	-3.48831370396188
C	-2.48265041429346	4.23368107593375	-3.19659451730498
H	-5.99571582394551	5.69954222722880	-0.80379051918720
H	-5.99847534412693	3.98715402934758	-1.32718290744596
H	-2.58497013515723	2.99866340169647	3.70759602924595
H	-4.03554125771120	2.30471562173069	4.50354540272089
H	-3.75100180571842	0.20613177091694	3.15577968471180
H	-2.36228544230774	0.56936773971589	4.23328894002585
H	0.30839742117515	-0.95317367032948	0.79886534729050
H	0.43568638902089	0.73391069295679	1.41156785555260
H	-1.03399895732889	0.47007632526461	-2.86291391671202
H	0.42152392353297	1.44961005430206	-3.22793897535793
H	-1.01451662472025	3.11637872110364	-4.28376398517082
H	-2.48525784683868	2.18258058550330	-3.86696231675075
H	-2.87578236206835	4.64430380791875	-4.14836780024283
H	-1.77168099411661	4.97065181978343	-2.77570624220634
H	-4.78682990745073	5.54690381375792	-2.88655692202095
H	-3.63341837259928	5.94230632793613	-1.57095181062580
H	0.82423742453848	1.71403781503071	-0.74698289085460
H	0.94136208325312	0.03865057319634	-1.36745065035423
H	-0.95489240069631	-0.71539203202673	2.85207566503677
H	-2.09768034865249	-1.01733763448401	1.50358206104820
K	-2.34008372440757	2.89600430766988	0.23934333757319
C	-4.77879275885789	4.00009662537608	2.59904153660699
C	-5.59425334622991	4.31749554413657	1.36594029729758
H	-3.97231959173658	4.74869915949998	2.71309753780725
H	-5.44176288194646	4.05030353477784	3.48637253213813
H	-6.13828367179743	5.26864964464030	1.53276455112865
H	-6.34480385381633	3.52194681100405	1.18679628229877
C	-0.24983931625046	-0.01003180257515	0.96769652003663
F	-0.39816680827351	4.00774583518956	1.36944174570385
F	5.16093198798655	10.30534504094966	2.54645095661006
H	7.00973939558240	8.51943911324812	2.28190854754625
O	5.55713048062758	8.40844502616787	4.43575007702973
C	6.34875152317804	7.64337409650773	2.35482647249871
C	4.38072670105577	9.19445761718360	2.51171036279753
H	6.88675090250103	6.83849341006978	2.87408934204112
H	4.84554893664823	8.75669342624973	5.06817219984372
C	5.09978371493552	7.98888543579286	3.18662107571615
H	6.08820581960786	7.29808359974936	1.34554925034690
F	3.22875311236199	9.51151485614937	3.11104251277085
F	4.10680097323035	8.98261272708622	1.20404048982256
H	4.52234630057176	6.46829634579212	5.37321202340169
C	4.18152513314880	6.74961667302168	3.26378854197945
C	4.05627737617358	6.06107123658219	4.47659961397718
C	3.54670548555506	6.20480376477578	2.13759012698516
H	3.60010467297950	6.70863038625147	1.17195304984301

C	3.36170215774056	4.85167027956845	4.55316500202347
H	3.31375639940116	4.34499035748489	5.51752917890759
C	2.83532022200445	5.00582690161960	2.21747322412777
C	2.74810200583623	4.29418969235469	3.42090147736053
H	2.35077613949980	4.61163105693338	1.32499874514154
F	1.58163263383733	2.83044160241839	5.79716202895703
F	0.06804609453916	3.63649068428454	4.47453145670660
C	2.10653601253616	2.88909667027674	3.42047945081618
H	3.75142107129595	1.99164609088170	4.57033748235883
C	1.05285795983084	2.72932379552254	4.55606811867939
C	3.20415850399903	1.83056833750753	3.63167126622171
H	3.91067775878913	1.90480100233105	2.79384839509469
H	0.72657847246280	3.22534078960410	1.95522622319405
O	1.47109814891681	2.58270324488093	2.21773863007003
F	0.46429521992539	1.51097995132681	4.50476724546737
H	2.76493826472742	0.82239888683127	3.63465481934972
F	-1.42043646952517	7.00331202589624	-0.27552125322511
H	-3.47141707405536	7.73527670582737	1.16263947777385
C	-0.78597255157034	7.50798317873901	0.81019924926578
O	-1.92343692178221	5.84251527802205	2.09803878118506
C	-2.90315618348738	7.98738265083143	2.07043012220700
F	0.44737423692706	6.99563372408006	0.81866709293852
F	-0.66362933793301	8.83753893932672	0.57685686363976
H	-1.17157715214861	5.19810302371634	1.87175014235317
C	-1.58200182302689	7.19647344338109	2.11121552442079
H	-3.49590270584353	7.69838360914864	2.94950831769253
H	-2.73155624825382	9.07145027760300	2.10738659542172
C	-0.79996643304874	7.59596907316998	3.38303078253291
H	-1.34138862858584	5.77661284605067	4.39348455814875
C	-0.84993963337488	6.74327590555239	4.49209081141558
H	-0.05981115406436	9.52022581005190	2.69956044061094
C	-0.13815420043383	8.82371081781352	3.53495486473376
C	-0.29033235100085	7.11122461072995	5.71747866278006
C	0.43673720390674	9.18480423434414	4.75570904228112
H	-0.37016910412915	6.41322642743099	6.55101718730346
C	0.35801269555636	8.34456137986389	5.87411080782514
H	0.95591736299960	10.13909851451032	4.84702622163488
C	0.87388704125355	8.87419764488762	7.23015732450616
F	2.43777905836528	7.03699609059796	7.42653965789627
H	2.69528787162370	9.60986161259853	6.67560032113747
F	0.60603980702081	6.86049676712519	8.56828623273367
O	1.81124547469664	9.89816468354082	7.08760881663991
C	1.51683348572783	7.74580516090519	8.09232402493920
H	-0.70224668732454	10.31620164190531	7.44699173712919
C	-0.31120193379919	9.46345691704645	8.01855423066130
H	-1.11696503521783	8.72933885657156	8.15245025160279
F	2.12894490732855	8.26126786391900	9.18225113391801
H	0.02312606540679	9.82303622299660	9.00335374648065
H	6.41312547499507	7.24058282219995	9.42595744773870
H	4.72542921945289	8.49358708517074	10.62993677742304
C	6.05200429776997	8.18805538334296	8.97817494365892
C	5.49325983848315	9.06736062557514	10.07549608436010

H	6.29479451898763	9.35136942565078	10.78688906807753
H	5.25261266458505	7.95573257070064	8.25031555519196
H	8.18252072097075	7.20829458196229	7.66971807618228
H	3.53397653174452	10.43025533194202	11.01970421927706
O	7.11803374315204	8.86506506406250	8.33584915121849
C	7.71649651073080	8.13627292957005	7.28099731665651
C	4.16095369393530	11.04130779006556	10.34154775690621
O	4.91857026167965	10.21324368512279	9.49024885377131
H	6.96360835367862	7.86065303180327	6.51929159388765
H	4.82634986068723	11.67145348019377	10.96594243861965
C	8.78749302145619	8.98776745851451	6.63467385088856
C	3.23970007455506	11.89555724296330	9.49940277596376
H	9.52300175482487	9.32395496975657	7.39254410771015
H	9.32324273132706	8.37344811099844	5.88432498343964
H	2.59759481586136	11.24200259588547	8.88121039295360
H	2.59753683271720	12.50176304550250	10.17019694143959
K	5.76580877973172	11.08878391937798	7.01383767538799
O	8.17535282944153	10.09524456393754	6.01035192945267
O	4.00575760713772	12.75126767647962	8.66142632714922
C	8.99616244679166	10.87622452593452	5.17596110411599
C	3.20914747438139	13.51108841493630	7.77558695068736
H	2.61086670146405	12.84247302768287	7.12800884144960
H	9.62970036631132	10.22918530089034	4.53746179522510
H	9.66783708195786	11.52232265307657	5.77642063696002
H	2.50539941406757	14.15283425337035	8.34385578501254
C	8.11935756263625	11.71558742125565	4.27214896501802
C	4.08982023791145	14.40267247344805	6.93010699306447
O	4.87955812346227	13.61334495361393	6.06475137149708
H	7.44339283022727	11.05255670840487	3.70127871810151
O	7.36346296216247	12.62693010533028	5.04885723960180
H	4.73757148858978	15.02286099666884	7.58084381911088
H	8.76061115716548	12.26547188836844	3.55392686726796
C	6.46286573221678	13.40641583583435	4.28726485782096
H	3.44453132721262	15.08579358676252	6.34218889694652
C	5.71266126076686	14.35134428352564	5.19815687089945
H	5.74882249122487	12.75306653302405	3.75093140780938
H	6.42917186317283	14.96373105228894	5.78065304150906
H	5.10711735754244	15.03902781334870	4.57415546498910
H	7.01259291909086	14.00365156180851	3.53213032011704
F	4.10066723051395	9.41186770254681	6.20543888526431
